# A High-Resolution Whole-Genome Map of Key Chromatin Modifications in the Adult *Drosophila melanogaster*

**DOI:** 10.1371/journal.pgen.1002380

**Published:** 2011-12-15

**Authors:** Hang Yin, Sarah Sweeney, Debasish Raha, Michael Snyder, Haifan Lin

**Affiliations:** 1 Yale Stem Cell Center and Department of Cell Biology, Yale School of Medicine, New Haven, Connecticut, United States of America; 2 Ottawa Hospital Research Institute, Ottawa, Canada; 3 Department of Molecular, Cell, and Developmental Biology, Yale University, New Haven, Connecticut, United States of America; The Babraham Institute, United Kingdom

## Abstract

Epigenetic research has been focused on cell-type-specific regulation; less is known about common features of epigenetic programming shared by diverse cell types within an organism. Here, we report a modified method for chromatin immunoprecipitation and deep sequencing (ChIP–Seq) and its use to construct a high-resolution map of the *Drosophila melanogaster* key histone marks, heterochromatin protein 1a (HP1a) and RNA polymerase II (polII). These factors are mapped at 50-bp resolution genome-wide and at 5-bp resolution for regulatory sequences of genes, which reveals fundamental features of chromatin modification landscape shared by major adult *Drosophila* cell types: the enrichment of both heterochromatic and euchromatic marks in transposons and repetitive sequences, the accumulation of HP1a at transcription start sites with stalled polII, the signatures of histone code and polII level/position around the transcriptional start sites that predict both the mRNA level and functionality of genes, and the enrichment of elongating polII within exons at splicing junctions. These features, likely conserved among diverse epigenomes, reveal general strategies for chromatin modifications.

## Author Summary

Just as a genome sequence map is indispensible to genetic studies, an epigenome map is crucial for epigenetic research. This is especially true for a sophisticated genetic model such as *Drosophila melanogaster*, where the wealth of information on genetics and developmental biology awaits systematic epigenetic interpretation on a whole-genome scale. In this manuscript, we report a high-resolution map of key chromatin modifications in the *Drosophila* genome constructed by the ChIP–Seq approach. This map is derived from all cell types in the adult *Drosophila* weighted by their natural abundance. It contains key histone marks, HP1a and RNA polymerase II, mapped at 50-bp resolution throughout the genome and at 5-bp resolution for regulatory sequences of genes. It reveals striking features of chromatin modification and transcriptional regulation shared by major adult *Drosophila* cell types. We anticipate that this map and the salient chromatin modification landscapes revealed by this map should have broad utility to the fields of epigenetics, developmental biology, and stem cell biology.

## Introduction

Epigenetics refers to the regulation of gene expression that is heritable to daughter cells without alteration of genetic information [1]. Epigenetic regulation is commonly achieved via DNA methylation, covalent modification of histones, and association/dissociation of chromatin factors [2]. Chromatin modifications of many genes in a genome in a specific fashion leads to epigenetic programming of the genome. It has been assumed that chromatin modifications occur in a cell-type-specific fashion in order to specify and maintain diverse cell fates [3]. This presumed central feature of chromatin modifications has been the subject of intensive investigation and has been supported by abundant evidence. However, of equal importance, there must also be common patterns of chromatin modifications that exist in all types of cells, which would reflect general features of the epigenome that are shared by diverse cell types within an organism or even among distant species. It is important to understand such general features of chromatin modifications, and substantial effort has been devoted to this area of study.

There is strong evidence supporting the existence of general features of chromatin modifications that are shared by all types of cells. Perhaps the strongest evidence is the presence of constitutive heterochromatin in centromeres and telomeres — a feature not only present in all types of nucleated cells within an organism but also well conserved during evolution [4]. Centromeric heterochromatin is essential for chromosome condensation and segregation during mitosis; whereas telomeric heterochromatin may be related to telomere function and telomeric silencing of transcription. Beyond these two examples, relatively little is known about the general features of chromatin modifications in the bulk of the genome, especially in the euchromatic genome. To explore these general features systematically, we combined high-resolution chromatin immunoprecipitation and high-throughput sequencing (ChIP-Seq) to map the distribution patterns of a panel of histone modifications, Heterochromatin Protein 1a (HP1a), and RNA polymerase II (RNA polII) in *Drosophila melanogaster*. This allowed us to construct a high resolution whole-genome map of *Drosophila* with these key chromatin modifications and the transcriptional activity mapped at 50 base-pair resolution. Our mapping data are consistent with recent major mapping efforts in *Drosophila* cell lines and major developmental stages [5], [6], [7], [8]. Moreover, our map, derived from all cell types in the adult *Drosophila* weighted by their natural abundance, reveals striking features of the chromatin modifications with important functional implications.

## Results

### A modified ChIP-Seq method that generates high-resolution whole-genome maps of chromatin modifications

**Figure 1:**
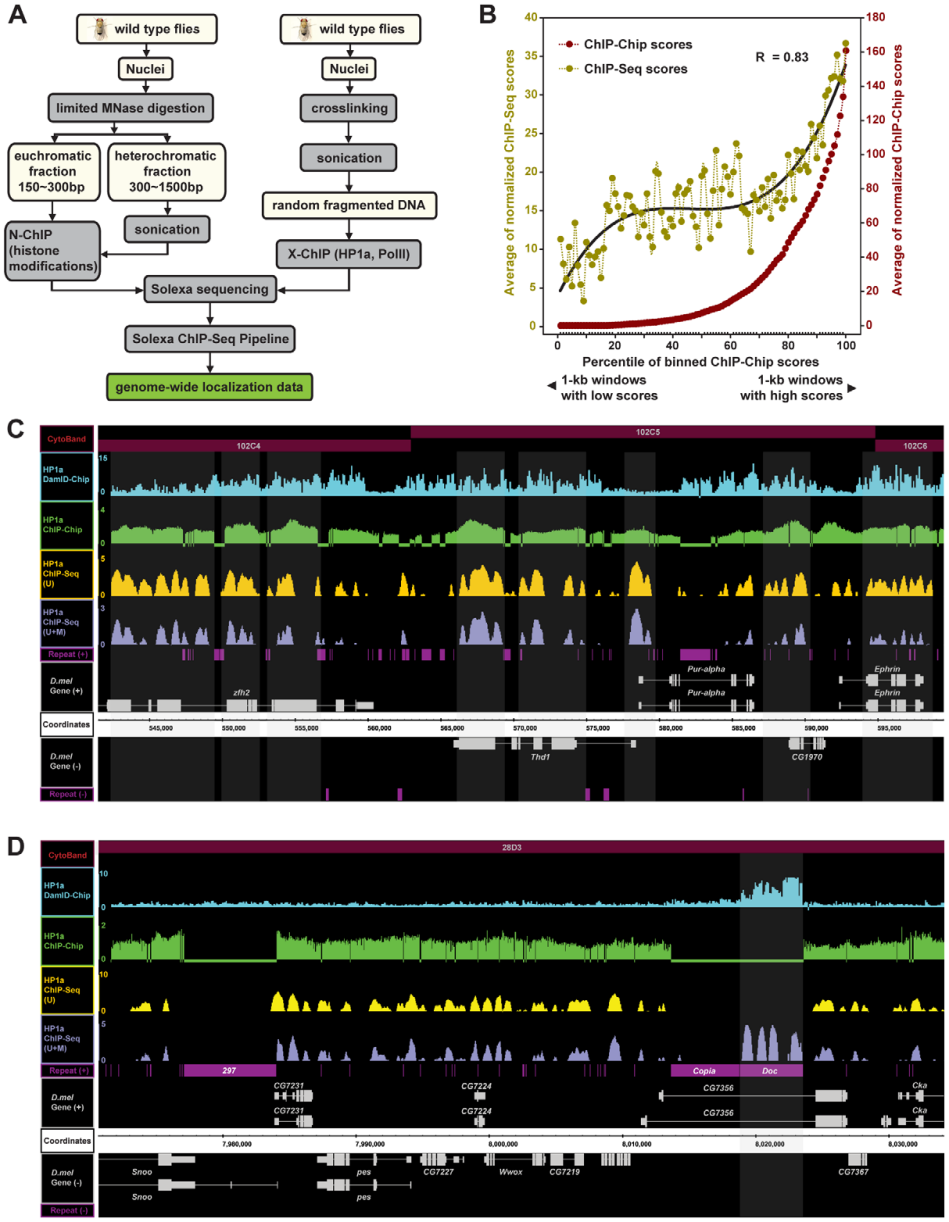
A modified ChIP-Seq method for high-resolution whole-genome mapping of chromatin modifications and the validation of the method. (A) The scheme of the modified high-resolution ChIP-Seq method. (B) A correlation analysis comparing maps of our ChIP-Seq method and ChIP-Chip [48] on HP1a localization in the wild type *D. melanogster* genome. 30,812 1-kb genomic regions interrogated by the ChIP-Chip analysis were ranked into 100 percentiles by their ChIP-Chip scores. Average scores for windows within a percentile were shown in dots for both ChIP-Seq (yellow) and ChIP-Chip (red). Because the ChIP-Chip assay does not include repetitive sequences, ChIP-Seq (U) scores were used in this analysis. Pearson Product-Moment correlation coefficient was calculated. (C) Comparison of HP1a localization within a 60-kb region on the 4^th^ chromosome by DamID-Chip (blue), ChIP-Chip (green), and our two ChIP-Seq analytical methods [ChIP-Seq (U) (yellow) and ChIP-Seq (U+M) (purple)]. The cytological bands (red), strand-specific gene features (grey) and strand-specific transposon/repetitive sequence features (magenta) are shown by different tracks. Shaded areas indicate transcription start sites (TSS) and exon-enriched regions, which are reproducibly identified as HP1a binding regions by multiple methods. (D) Comparison of HP1a distribution within a 64-kb genomic region on chromosome 2L by DamID-Chip (blue), ChIP-Chip (green) and our two ChIP-Seq analytical methods [ChIP-Seq (U) (yellow) and ChIP-Seq (U+M) (purple)]. Shaded areas indicate HP1a peaks identified previously at a *Doc* retrotransposon [48].

To gain high resolution whole-genome maps of the *Drosophila* chromatin modification, we isolated nuclei from whole adult flies for ChIP-Seq. In order to achieve an unbiased representation of both euchromatin and heterochromatin in the following ChIP, we modified the standard ChIP-Seq method by first treating nuclei with limited amount of micrococcal nuclease (MNase) and then separating chromatin into euchromatic and heterochromatic fractions (Figure 1A). Chromatin in heterochromatin fractions was further fragmented by sonication into a size range comparable to the euchromatic chromatin (Figure S1A). Chromatin from euchromatic and heterochromatic fractions were subjected to immunoprecipitation of post-translationally modified histone 3: histone 3 trimethylated at Lysine 4 (H3K4me3) and acetylated at lysine 9 (H3K9ac) as euchromatic marks, whereas histone 3 trimethylated at Lysine 9 (H3K9me3) and trimethylated at Lysine 27 (H3K27me3) as heterochromatic marks. To minimize biases introduced by partial MNase digestion and nucleosome positioning, we preformed the immunoprecipitation of total histone 3 (H3) as a control for normalization. In addition, crosslinked chromatin was used for immunoprecipitation of HP1a, a heterochromatic protein, as well as RNA polII that indicates transcription activity (Figure 1A). For these two epigenetic marks, a mock ChIP was conducted as a control for normalization. The high specificity of HP1a antibody used in this study was confirmed by Western blotting (Figure S1B). All precipitated DNA was sequenced by Illumina Genome Analyzer 1G, which achieved 7.9-fold coverage of the *Drosophila* genome in total (Table S1). The relative abundance of epigenetic marks across the entire genome was quantified as detailed in Materials and Methods and Figure S1.

So far, most published bioinformatic analyses of ChIP-Seq are based exclusively on unique-mapping (i.e. deriving from single genomic location) Illumina reads, which have unambiguous genomic origins [9]. However, we find that ∼24.5% of Illumina reads from the mock ChIP sample are multiple-mapping reads with more than one matching site within the genome (Table S1). BLAST analyses indicate that these multiple-mapping reads represent repetitive, low complex, and transposon-derived sequences, frequently found in heterochromatic regions of the *Drosophila* genome (data not shown). The fact that some heterochromatic marks are mostly enriched in repetitive sequences and that these repetitive sequences function in heterochromatic silencing demands the inclusion of these multiple-mapping reads in the ChIP-Seq analyses. To this end, we employed two different calculations in the score generation step of ChIP-Seq analyses: a unique-mapping only method, which calculates the ChIP-Seq scores purely based on unique-mapping reads [ChIP-Seq (U)]; and a method combining both unique-mapping and multiple-mapping reads [ChIP-Seq (U+M)] (Figure S1). In the latter method, a multiple-mapping tag contributes equally to all matching genomic sites with score matrices weighted by the reciprocal of the number of genomic matching sites. Although this method cannot discriminate multiple matching sites for a single Illumina read, we reasoned that many multiple-mapping reads and unique-mapping reads together will generate individual scores for similar transposon/repetitive sequences in the genome. A similar approach was recently employed to interrogate H3K9me3 distribution pattern within repetitive genomic regions in human CD4+ T lymphocytes [10].

To validate our ChIP-Seq analyses, we first compared our ChIP-Seq results of HP1a distribution patterns with the published results of HP1a Chromatin IP combined with the genome tiling array experiment (ChIP-Chip) in *Drosophila* S2 cells [11] and DNA adenine methyltransferase identification combined with the genome tiling array experiment (DamID-Chip) in adult whole flies [12]. Our ChIP-Seq (U) results faithfully reproduce HP1a localizations from the ChIP-Chip assay with a Pearson Product-Moment correlation coefficient as high as 0.83 (Figure 1B). We find that both ChIP-Seq (U) and ChIP-Seq (U+M) results feature eminent resolutions and can largely replicate previous observations of HP1a distributions in a gene-rich region (Figure 1C). Strikingly, our ChIP-Seq (U+M) scores successfully recapitulate previous findings of the DamID-Chip assay showing that HP1a is specifically associated with a *Doc* retrotransposon, but not with an adjacent *copia* retrotransposon (Figure 1D). Overall, our ChIP-Seq (U+M) results largely repeat the HP1a distribution patterns from DamID-Chip assay (Pearson correlation coefficient = 0.77, Figure S2). Again, our ChIP-Seq (U+M) data on HP1a features much higher resolution (50 bp) as compared to the DamID-Chip method.

### Whole-genome mapping reveals strikingly distinct distribution patterns of chromatin modifications with respect to specific types of genomic sequences

**Figure 2:**
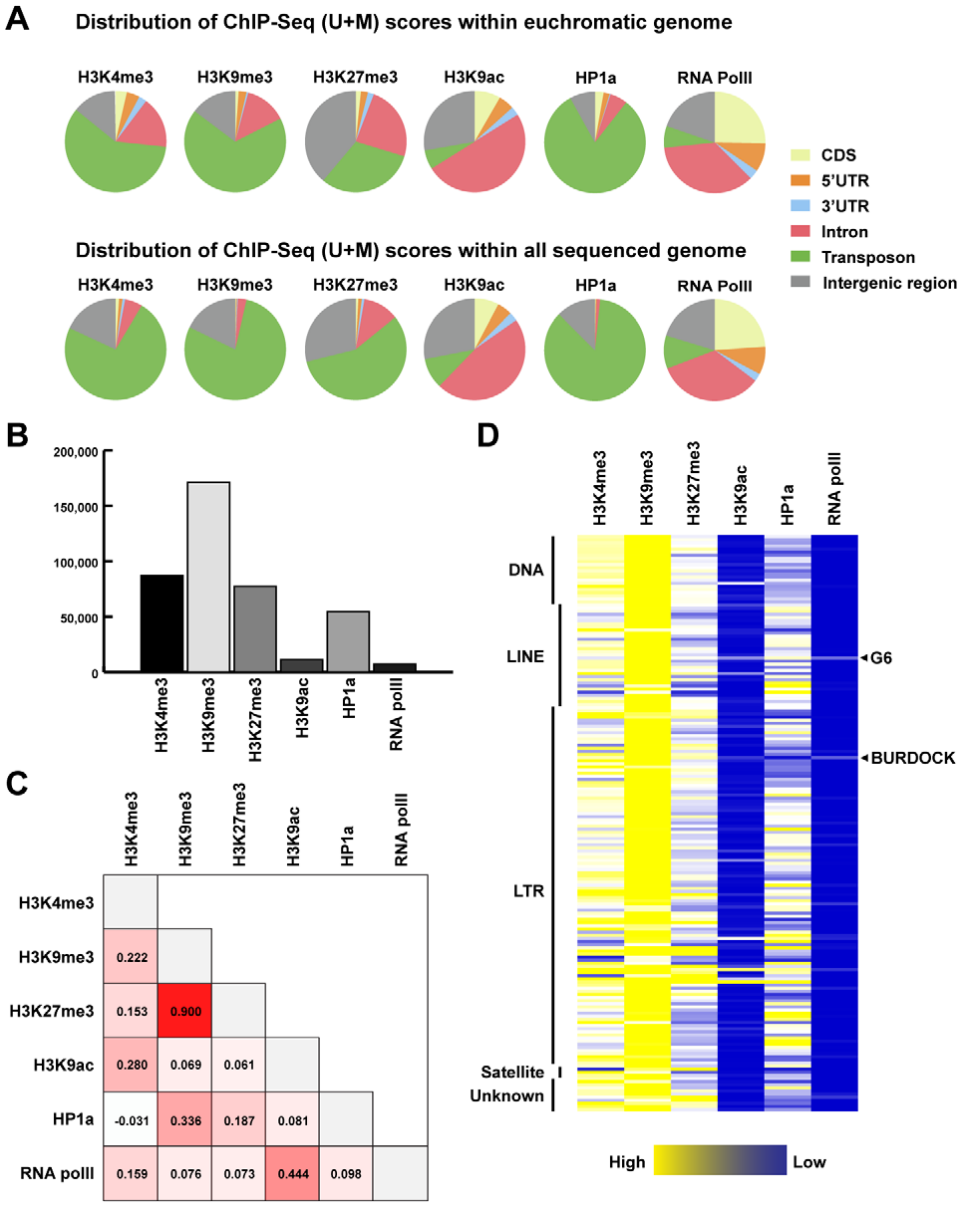
Distribution of Illumina tags and ChIP–Seq (U+M) scores over main features of the genome. (A) Distribution of ChIP-Seq (U+M) scores over CDS, 5′ UTR, 3′ UTR, intronic, transposon/repetitive and intergenic regions within euchromatic genome and all sequenced genome. (B) Total ChIP-Seq (U+M) scores of epigenetic marks in all transposons within the genome. (C) Pearson correlations between ChIP-Seq (U+M) scores of epigenetic marks over 185 transposon classes. (D) A heat map of ChIP-Seq (U+M) scores of epigenetic marks over 185 transposon classes, which are arranged into 5 major types: DNA, LINE, LTR, Satellite and unknown.

Using the above-described method, we conducted the whole-genome mapping of H3K4me3, H3K9me3, H3K27me3, H3K9ac, HP1a, and RNA polII in euchromatic arms (chrX, chr2L, chr2R, chr3L, chr3R and chr4; hereafter called euchromatic genome) as well as other sequenced internal scaffolds and unmapped regions (XHet, 2LHet, 2RHet, 3LHet, 3RHet, YHet, U and Uextra; hereafter called heterochromatic genome). To gain an overview of the distributions of chromatin modifications, we compared their ChIP-Seq (U+M) scores over different genomic features (CDS, 5′UTR, 3′UTR, intron, transposon/repetitive sequence, and intergenic region) within the euchromatic genome and all sequenced genome (Figure 2A). This comparison reveals distinct distribution patterns of chromatin modifications in the genome related to specific types of genomic sequences. We find RNA polII and H3K9ac are highly enriched in protein-coding genes, with 69.3% of RNA polII scores and 62.3% of H3K9ac scores located within CDS, 5′UTR, 3′UTR and intron regions (Figure 2A). This is consistent with the notion that these two chromatin modifications are associated with actively transcribing genes [13]. Within genes, RNA polII and H3K9ac show distinct distribution patterns with respect to subgenic regions: RNA polII is preferentially present in CDS and 5′UTR regions whereas H3K9ac is relatively enriched in introns. In contrast to these euchromatic marks, 85.9% of HP1a scores and 78.7% of H3K9me3 scores are situated in transposons and repeats within all sequenced genome (Figure 2A), which largely reflect the natural abundance of these two marks on polytene chromosomes [14]. Interestingly, we find transposons and repeats include 59.3% and 73.3% of H3K4me3 scores within euchromatic and all sequenced genome, respectively (Figure 2A). This is consistent with previous reports that both euchromatic (H3K4me3) and heterochromatic (H3K9me3) marks are present within heterochromatin [15], [16].

To explore the chromatin modification of transposons, we calculated the total ChIP-Seq (U+M) scores of chromatin modifications on all transposons in the genome. We find that heterochromatic marks H3K9me3, H3K27me3 and HP1a are abundant within transposons (Figure 2B). In contrast, transposons are mostly devoid of transcription activity marks, H3K9ac and RNA polII. These results are consistent with the notion that most transposons in the *Drosophila* genome are transcriptionally silenced whereas a small portion of transposons remain transcriptionally active [17]. To investigate epigenetic marks co-localized in transposons, we performed pair-wise Pearson correlation analyses for chromatin modification densities in transposons classified into 185 classes (Figure 2C). The significant positive correlation between H3K9me3 density and H3K27me3 density indicates these two chromatin modifications are co-localized on transposons (P.c. = 0.9, *p* = 8.746×10^−68^). We find H3K9me3 is also co-occcurring with HP1a within transposons (P.c. = 0.336, *p* = 3.01×10^−6^), which suggests HP1a is recruited here by this mark. In addition, correlated RNA polII and H3K9ac densities (P.c. = 0.444, *p* = 2.42×10^−10^) implicates some transposons, like *G6* and *Burdock*, are transcriptionally active in the *Drosophila* genome (Figure 2C, 2D).

### Euchromatic marks H3K4me3 and H3K9ac in active genes are enriched in the transcription initiation sites and throughout the transcription unit, respectively

**Figure 3:**
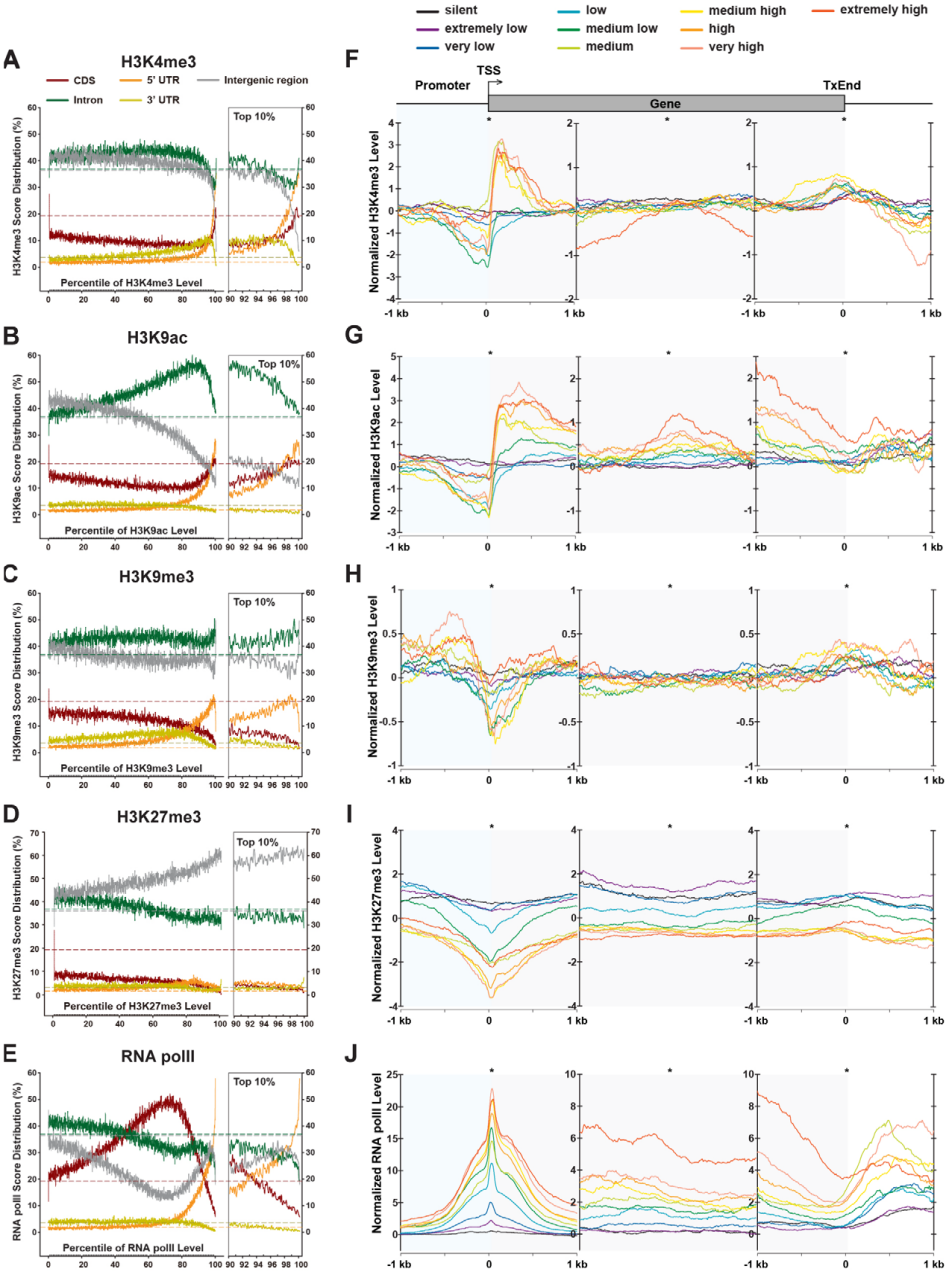
Distributions of chromatin modifications across the genome and genes. (A–E) Distribution of H3K4me3, H3K9me3, H3K27me3, H3K9ac and RNA polII ChIP-Seq scores across the genome. The genome was divided into 2,407,635 50-bp windows, which were ranked into 100 percentiles based on the ChIP-Seq scores of chromatin modifications (X axis). For each percentile, the percentages of windows overlapping with 5′ UTR, CDS, intron, 3′ UTR, and intergenic regions were drawn in different colors. The percentages of these genomic features in the genome are indicated by dash lines in the corresponding colors. The right panel shows detailed distribution profiles for the top 10 percentiles. (F–J) ChIP-Seq score profiles of H3K4me3, H3K9me3, H3K27me3, H3K9ac and RNA polII surrounding protein coding genes. 20,738 transcripts from 9,338 protein coding genes were aligned at transcription start sites, mid points of transcript, and transcription end sites. Distribution of ChIP-Seq scores within 2-kb regions around the aligning points were interrogated in 10-bp resolution. Genes were classified into 10 groups based on their expression levels.

**Figure 4:**
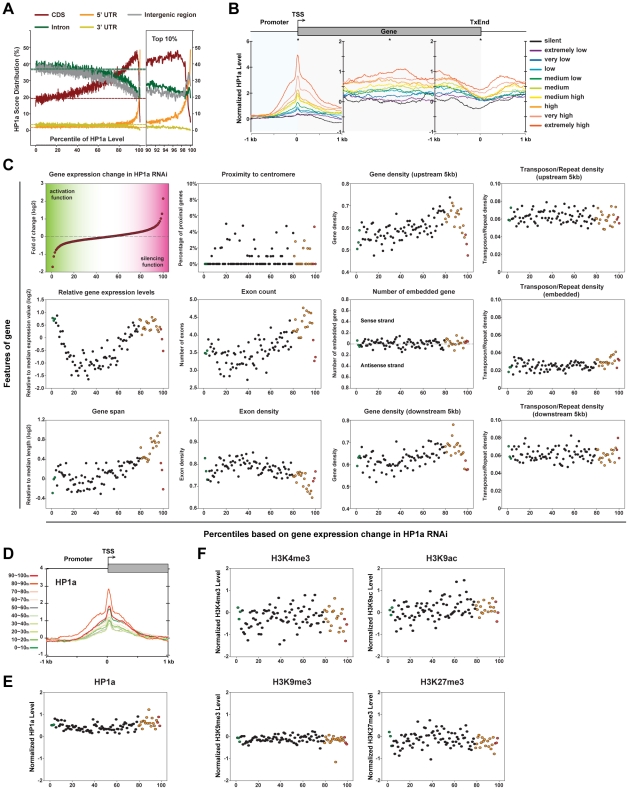
HP1a localizes at TSSs and its functions in both activation and silencing. (A) Distribution of HP1a ChIP-Seq scores across the genome (see detailed description in Figure 3 legend). (B) The ChIP-Seq score profile of HP1a surrounding protein coding genes (see detailed description in Figure 3 legend). (C) The correlations between various features of protein coding genes and gene expression changes in HP1a knockdown [29]. Averaged fold of change in gene expression together with 11 other features of protein coding genes (Y-axles) were plotted for 100 percentiles of genes (X-axles) sorted and grouped by folds of change in gene expression (fold of change percentile; see Method section for details in calculation). For the number of embedded genes in gene body, embedded genes on both sense strands (upper) and antisense strands (lower) were calculated separately. Only the dominant values were plotted. (D) The ChIP-Seq score profile of HP1a surrounding the TSSs of protein coding genes grouped by percentiles in folds of change in gene expression. (E) HP1a levels around TSSs (+/−500 bp) for genes in 100 fold of change percentiles. (F) Levels of histone modifications around TSSs (+/−500 bp) for genes in 100 fold of change percentiles.

To further investigate the enrichment patterns of chromatin modifications within protein-coding genes, we sorted ∼2.4 million 50-bp windows within euchromatic genomes into 100 percentiles based on their ChIP-Seq (U) scores and calculated the percentages of genomic features for every percentile individually (Figure 3A–3E, Figure 4A). The relative abundance of a chromatin modification over a genomic feature was determined by comparing the percentages to the natural representation of the genomic feature within the euchromatic genome (Figure 3A–3E, Figure 4A). Furthermore, we determined the distribution of these chromatin modifications relative to the transcriptional start sites (TSSs), the mid points of gene bodies, and the transcription end sites (TxEnds) of protein coding genes with regard to their transcriptional levels (Figure 3F–3J, Figure 4B). 6,756 genes with known gene expression levels were classified into 10 groups according to their relative expression levels in whole fly samples interrogated by microarray experiments (GSE5382, GSE7763), with each group representing a 10% increment of expression levels.

Within protein coding genes, the top 10% of H3K4me3- and H3K9ac-dense sequences are highly represented in 5′UTRs and CDSs (Figure 3A and 3B). Specifically, both H3K4me3 and H3K9ac are highly enriched in the 5′ ends of high- and medium-expressing genes (+50 bp∼+750 bp for H3K4me3 and +50 bp∼+1 kb for H3K9ac), but sharply declined around TSSs (−50 bp∼+50 bp) and severely under-represented in proximal promoter regions (−600 bp∼TSS) (Figure 3F and 3G). Such a dynamic pattern is not observed in low-expressing and silent genes.

H3K9ac differs from H3K4me3 in two additional features within protein coding genes. First, moderately to highly H3K9ac-dense sequences (70^th^∼90^th^ percentiles) are also enriched in intronic sequences but devoid from intergenic regions. This is consistent with the notion that H3K9ac specifically associates with transcriptional activity and can spread over the whole gene body [18]. Second, H3K9ac is enriched in 3′ends of genes (−1 kb regions upstream of TxEnds) of medium- and high-expressing genes, in contrast to the slight enhancement of H3K4me3 at the TxEnds (Figure 3F and 3G).

### Heterochromatic marks H3K9me3 and H3K27me3 are enriched in transposons and repetitive sequences

The H3K9me3 mark is the binding target of HP1a, and is generally regarded as an epigenetic silencing mark [19], [20]. Within protein coding genes, extremely H3K9me3-dense (top 2%) sequences are located in intergenic and intronic regions (Figure 3C). Intriguingly, in actively transcribed genes, H3K9me3 is highly enriched in the promoter region (−1 kb∼−100 bp) but generally depleted in the 5′ ends of genes (Figure 3H). This pattern is opposite to that of H3K4me3 and H3K9ac, and echoes recent observations that H3K9me3 is associated with promoters of active genes in mammalian genomes [21], [22].

Similarly, H3K27me3, the binding target for Polycomb repressive complex 1 (PRC1), is enriched in discrete intergenic regions (Figure S3A, Table S2), but under-represented in CDS, 3′UTR and intronic regions (Figure 3D). Most of H3K27me3-enriched regions are located within cytological bands that were previously identified as cytobands bound by Polycomb proteins on polytene chromosomes and S2 cells (Figure S3A) [5], [8], [23], [24], [25]. Moreover, of 167 predicted PRE/TREs [25], 89 are enriched for the H3K27me3 marks, which validates these PRE/TRE as constitutive binding sites for PRC1 in adult flies. For example, the three most prominent H3K27me3-enriched regions on chromosome arm 3R are the *Antennapedia* complex (*ANT-C*), *Bithorax* complex (*BX-C*), and a 200-kb region between *mod(mdg4)* and *InR*, which contains multiple predicted PRE/TREs (Figure S3B). At boarders of *ANT-C* and *BX-C*, as well as in active genes *CG7922* and *CG7956*, H3K27me3 is dramatically reduced to background levels. On average, genomic regions surrounding the 167 predicted PRE/TREs are significantly enriched for H3K27me3 marks comparing to randomly selected intergenic regions within the euchromatic genome (Figure S3C). Expectedly, the density of H3K27me3 in the promoter, 5′ ends, bodies, and 3′ ends of protein coding genes are negatively correlated to mRNA levels (Figure 3I). H3K27me3 is generally absent from medium- and high-expressing genes, but is enriched in the promoters and 5′ ends (−1 kb∼+1 kb) of silent and extremely low-expressing genes. This pattern resembles the distribution of H3K27me3 in the human genome [26] and reflects its function in long-term gene silencing [24], [27]. Notably, for low expressing genes, H3K27me3 is enriched in the promoter regions (−1 kb∼−250 bp) and 5′ ends (+200 bp∼+1 kb), but is absent around the TSSs. This observation appears to be consistent with recent findings that H3K27me3 and H3K4me3 are co-localized at a group of ‘bivalent’ promoters poised for transcription [28].

### RNA polymerase II level in a gene strictly corresponds to its RNA expression level

Consistent with the fact that RNA polII is the central player of transcription, the top 20% of polII-dense sequences are conspicuously over-represented within 5′UTRs and intergenic regions, yet moderately polII-dense sequences (within 40∼80%) are also enriched in CDS (Figure 3E). Moreover, the level of RNA polII is strictly correlated to the RNA expression level (Figure 3J). Particularly, polII concentrates around TSSs, forming a sharp peak within a narrow region immediately downstream of TSSs (0 bp∼+100 bp, Figure 3J). Significant RNA polII signals are also present within gene bodies and at the 3′ ends of expressing genes.

### HP1a localizes at TSSs of active genes and has both activating and silencing functions

Although HP1a is predominantly associated with transposons and repeats, about 23% of HP1a ChIP-Seq (U+M) scores are present in genic/intergenic regions. Within these regions, HP1a is particularly enriched in the 5′UTR regions and coding sequences (Figure 4A). Within a transcriptional unit, HP1a is highly concentrated around the TSS with only low levels of HP1a spreading over the gene body (Figure 4B). Strikingly, the levels of HP1a concentration at the TSSs are strictly correlated to the mRNA levels of its residing genes, confirming previous reports (see Discussion). Particularly, the sharp peaks of HP1a immediately surrounding TSSs (0 bp∼+100 bp) mimic the polII enrichment within the same regions. These prominent similarities strongly suggest HP1a functions together with RNA polII in transcription (see Discussion).

The high levels of agreement between our whole-fly-derived HP1a scores and ChIP-Chip scores generated from embryonic S2 cells indicate HP1a localizations are generally stable during development. Thus, we recruited a published microarray dataset, which contains gene expression data for both wild type third instar larva with and without HP1a-knockdown [29]. 12,521 interrogated genes were sorted and grouped into 100 percentiles based on their folds of changes in gene expression (hereafter called fold of change percentiles; Figure 4C).

To better understand genes regulated by HP1a, we calculated 11 additional features for genes in all percentiles (Figure 4C). Interestingly, genes highly repressed in HP1a RNAi knockdown larva (1^st^∼3^rd^ fold of change percentiles, green dots) are overly high-expressing genes in wild type larva, which are generally short in length and away from centromeres. By contrast, genes highly activated by HP1a knockdown (97^th^∼100^th^ fold of change percentiles, red dots) are generally devoid of any recognizable feature. We find a distinct third class of genes, representing moderately activated genes in HP1a knockdown (80^th^∼97^th^ fold of change percentiles, yellow dots). This class predominantly contains high-expressing, large genes, characterized by their large numbers of sparsely located exons. Notably, these genes also tend to localize within gene-rich regions. However, none of the above gene classes is correlated to transposon/repeat densities either upstream, downstream or within the gene bodies.

The above analyses implicate that HP1a concentrated at TSSs may have a direct function in regulating the expression of its target genes. To understand this function, we asked whether HP1a is specifically enriched at TSSs of its target genes. The HP1a density surrounding TSSs of 10 gene classes grouped by 10% increments of fold of change percentiles was investigated (Figure 4D). We find HP1a is enriched at TSSs of genes that are either highly repressed (1∼10% percentile) or highly activated (90∼100% percentile) in HP1a knockdown, indicating HP1a has direct functions of both activation and silencing on its target genes. Intriguingly, the highest levels of HP1a enrichment at TSSs are found among the third class genes that are moderately activated by HP1a RNAi, suggesting this gene class represents a distinct HP1a-mediated regulome.

We further calculated averaged levels of histone modifications over TSS regions (+/−500 bp) for all percentiles but failed to identify any correlation (Figure 4E–4F). This suggests that HP1a-mediated gene expression regulation is globally independent of other examined chromatin modifications.

### Stalled and elongating RNA polymerase II are positioned at +35 bp and +45 bp, respectively

**Figure 5:**
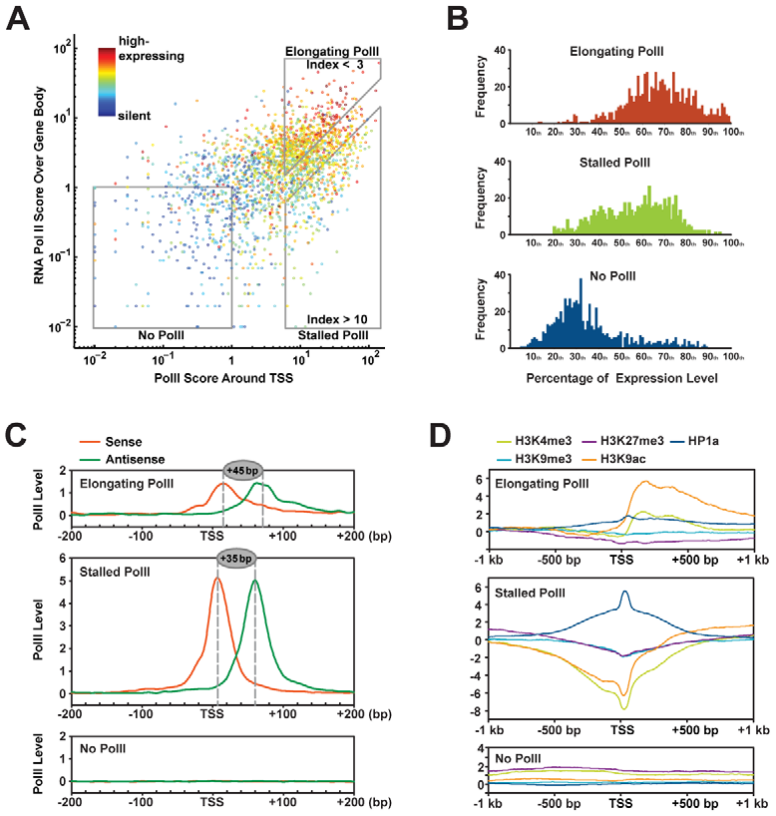
Correlation between chromatin modifications at TSS and gene expression. (A) RNA polII scores around TSS (X axis) and over gene bodies (Y axis) of 9,338 protein-coding genes correlate to their relative expression levels (color spectrum). Genes with no RNA polII, stalled polII, and elongating polII are grouped into 3 boxes. (B) Expression profiles of genes with elongating polII, stalled polII, and without polII. (C) RNA polII positions at the transcription start sites of genes with elongating polII, stalled polII and genes without polII. Tag mapped to the sense strands and antisense strands are separately plotted for their densities around TSS. (D) Distributions of chromatin modifications around TSS in genes with elongating polII, stalled polII and without polII.

Recent studies have revealed that RNA polII is poised or stalled at the TSS regions of about 10% genes in the *Drosophila* genome [30], [31]. It has been proposed that these poised/stalled polII allow rapid responses of gene activation to environmental stimuli and developmental cues. To gain a detailed view of RNA polII dynamics and gene expression, we adopted a previously established strategy [31] and categorized TSSs of genes into three classes: those with elongating polII (785 TSSs), stalled polII (685 TSSs) or no polII (695 TSSs; Figure 5A). Notably, stalled polII is detected in the TSS of *Hsp70* gene (CG18743), which is the first defined gene with stalled polII [32]. We find that the presence of elongating polII at the TSSs corresponds to genes within the top 50% expression levels whereas absence of polII at TSSs represents genes within the lowest 40% expression levels (Figure 5B, upper and lower panels). Interestingly, genes with stalled polII at their TSSs exhibit a broader range of expression levels (Figure 5B, middle panel).

To infer the precise positions of RNA polII at different types of TSSs, we calculated the frequency of polII-immunocoprecipitated reads matched to the sense and the antisense strands of genes and binned these reads into 5-bp windows (Figure 5C). A similar approach has been previously employed to position nucleosomes surrounding TSS regions [33]. By this method, we pinpoint stalled polII into a narrow region, centered at the +35 bp position (Figure 5C, middle panel). This location is identical to previous permanganate footprinting results, which localized open transcription bubbles within this region [31]. In contrast, for genes with elongating polII, only 30∼40% of polII resides around the TSS, however, it resides at the +45 bp position (Figure 5C, upper panel). The Kolmogorov-Smirnov test confirms that both of the 5′ ends distribution and the 3′ ends distribution of polII-immunocoprecipitated reads between stalled polII group and elongating polII group are statistically significant (5′ end: *p* = 2.3×10^−3^; 3′ end: *p* = 1.7×10^−4^). This 10-bp difference of RNA polII position may reflect distinct pausing stages during the transition from transcription initiation to fully engaged elongation. It may be used as a signature to predict the transcriptional activity of a gene.

### TSSs of actively transcribed genes have distinct signatures of chromatin modifications that reflect gene function

To understand the relationship between RNA polII stalling and epigenetic regulation, we analyzed the distribution of chromatin modifications within 2-kb regions around different classes of TSSs (Figure 5D). Interestingly, polII-stalled TSSs are associated with a strong peak of HP1a but not other chromatin modifications (Figure 5D, middle panel). This echoes our finding that HP1a-mediated gene expression regulation is independent of other interrogated chromatin modifications and suggests that HP1a is not recruited here by H3K9me3, but possibly rather by interaction with RNA polII. Distinct to this profile, genes with elongating RNA polII show very low levels of HP1a at TSSs but high levels of H3K4me3 and H3K9ac downstream of TSSs (Figure 5D, upper panel).

**Figure 6:**
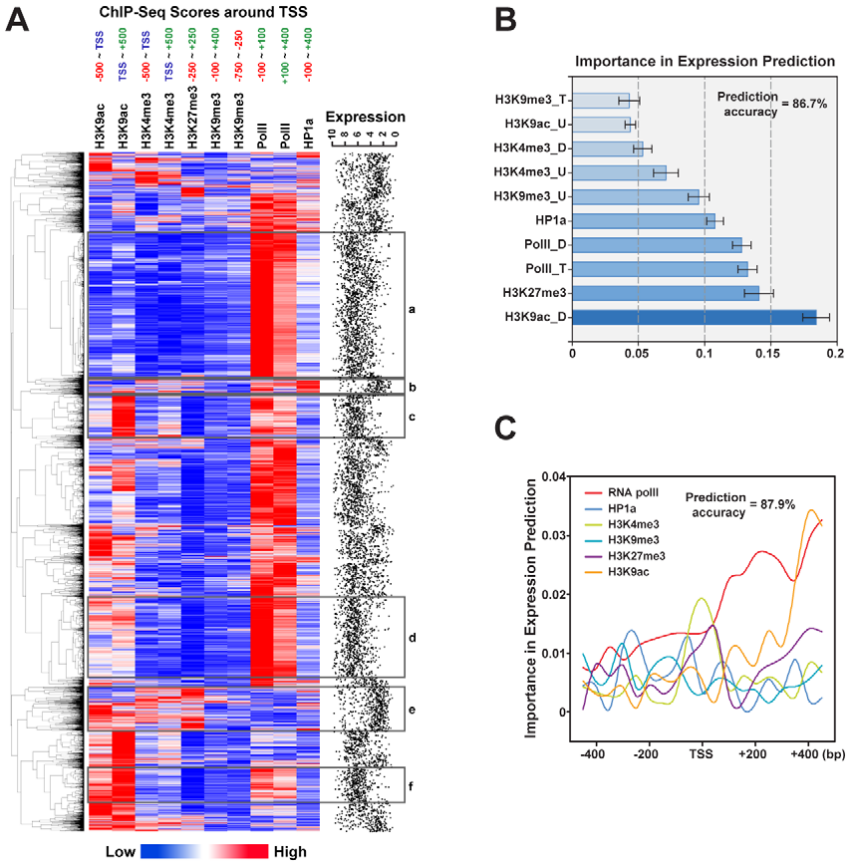
Epigenetic signatures around the TSS can predict gene expression levels. (A) Supervised hierarchical clustering of protein coding genes based on ChIP-Seq scores of epigenetic regulators/marks around their TSSs. The relative expression levels of clustered genes were shown on the right in a scale of 0 (not expressed) to 10 (highly expressed). Genes with similar epigenetic profiles at TSSs were grouped and shown in rectangles. (B) Prediction of gene expression levels by dynamic artificial neural network (ANN). The histogram shows relative importance of individual input variables (epigenetic regulators/marks) in the prediction. Error bars indicate the standard deviations from 10 runs of independent predictions. H3K4me3_U: averaged score of H3K4me3 upstream of TSSs (−500 bp∼TSS). H3K4me3_D: averaged score of H3K4me3 downstream of TSSs (TSS∼+500 bp). H3K9me3_T: averaged score of H3K9me3 around TSSs (−100 bp∼+400 bp). H3K9me3_U: averaged score of H3K9me3 upstream of TSSs (−750 bp∼−250 bp). H3K9ac_U: averaged score of H3K9ac upstream of TSSs (−500 bp∼TSS). H3K9ac_D: averaged score of H3K9ac downstream of TSSs (TSS∼+500 bp). H3K27me3: averaged score of H3K27me3 around of TSSs (−250 bp∼+250 bp). HP1a: averaged score of HP1a around of TSSs (−100 bp∼+400 bp). polII_T: averaged score of RNA polII around TSSs (−100 bp∼+100 bp). polII_D: averaged score of RNA polII downstream of TSSs (+100 bp∼+400 bp). (**C**) Prediction of gene expression levels by dynamic artificial neural network (ANN). The curves show relative importance of epigenetic regulators/marks at 19 positions around TSSs (−450 bp∼+450 bp, in 50-bp steps) in the prediction.

To further explore the overall effect of chromatin modification on gene expression, we clustered 7,826 *Drosophila* genes with known expression levels based on similarities of their epigenetic profiles around TSSs (Figure 6A). Interestingly, hierarchical clustering reveals six prominent gene clusters, each of which displays a characteristic gene expression profile and epigenetic signature around TSSs. Cluster (a) represents high-expressing genes with only high levels of RNA polII but no other epigenetic marks. Gene ontology analysis indicates this cluster is enriched for genes involved in transcription regulation, alternative splicing and development (Table S3). Cluster (b) contains low-expressing/silent genes with medium levels of RNA polII and H3K27me3 but high levels of HP1a. Cluster (c) and (d) consist of high-expressing genes with high levels of RNA polII, and high levels and medium levels of H3K9ac, respectively. These clusters are enriched for housekeeping genes, related to ribosome functions. Cluster (e) represents low-expressing/silent genes with H3K4me3, H3K27me3 and H3K9ac present at TSSs. This cluster is enriched for genes involved in G-protein coupled receptors. Cluster (f) contains medium-expressing genes with medium levels of RNA polII and high levels of H3K9ac. This cluster is enriched for oxidoreductases encoding genes. The above data reveal strong correlations between histone codes surrounding TSSs and expression of genes with distinct types of biological functions in a whole organism context.

To further understand this correlation, we employed a four-layer artificial neural network (ANN) [34] to predict gene expression levels by quantitative values of chromatin modifications around TSSs. With 50% of data allocated as a training set, we achieved 86.7% accuracy in the prediction of quantitative gene expression levels, which strongly suggests a causal relationship between TSS-located histone codes and gene expression (Figure 6B). Furthermore, we extracted weights for an individual “neuron” within the input layer after training, and identified H3K9ac downstream of TSSs and H3K27me3 surrounding TSSs as the two most critical factors determining the accuracy of target gene expression prediction (Figure 6B). To further narrow down the critical regions of these chromatin modifications in determining gene expression, we fed a neural network with averaged densities of chromatin modifications in nineteen 50-bp windows around TSSs (−450 bp∼+450 bp). With overall 87.9% accuracy, we find the presence of RNA polII and H3K9ac downstream of TSSs (0∼450 bp) are remarkable positive predictors of gene expression (Figure 6C). In addition, H3K4me3 and H3K27me3 around TSSs (−100 bp∼+100 bp) are also pivotal to gene expression prediction, which echoes the opposing functions of Trithorax group proteins (TrxG) and Polycomb group proteins (PcG) in regulating gene expression.

### RNA polymerase II is enriched on the exon sides of exon-intron and intron-exon junctions

**Figure 7:**
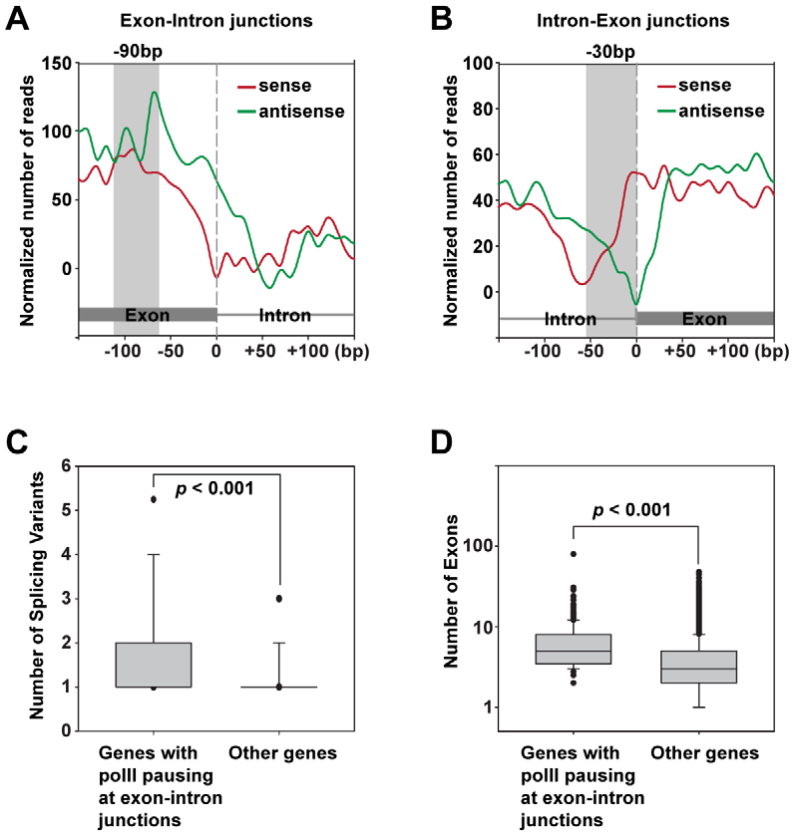
Enrichment of RNA polII on the exon sides of exon-intron and intron-exon junctions. (A) Enrichments of RNA polII signal at the exon side of exon-intron junctions. Normalized numbers of RNA polII Illumina reads mapped to sense and antisense strands were plotted separately. Shaded area indicates a polII-enriched region, centered at −90 bp upstream of exon-intron junctions. (B) Enrichments of RNA polII signal at the exon side of intron-exon junctions. Shaded area indicates RNA polII is devoid from a region centered at −30 bp upstream of intron-exon junctions. (C) A floating box graph comparing numbers of splicing variants for 254 genes with RNA polII pausing at exon-intron junctions with remaining genes in the *Drosophila* genome. The upper and lower boarders of the boxes indicate 75^th^ and 25^th^ percentiles, respectively. The upper and lower bars indicate 90^th^ and 10^th^ percentiles, respectively. Black dots are outliers beyond the 90^th^ and 10^th^ percentiles. The *p* value was calculated by Mann-Whitney Rank Sum test as both datasets failed a normality test (*p*<0.05). (D) A floating box graph comparing numbers of exons for 254 genes with RNA polII pausing at exon-intron junctions with remaining genes in the *Drosophila* genome. The upper and lower boarders of the boxes indicate 75^th^ and 25^th^ percentiles, respectively. The upper and lower bars indicate 90^th^ and 10^th^ percentiles, respectively. Black dots are outliers beyond the 90^th^ and 10^th^ percentiles. The *p* value was calculated by Mann-Whitney Rank Sum test as both datasets failed a normality test (*p*<0.001).

In searching for chromatin modifications at exon-intron and intron-exon junctions, we discovered that RNA polII is unevenly distributed at splicing junctions. Specifically, RNA polII is concentrated within exons with a prominent peak centered at −90 bp upstream of exon-intron junctions (Figure 7A). By contrast, RNA polII scores drastically drop to the background levels once the transcription machinery goes into introns. At intron-exon junctions, RNA polII is devoid from the region centered at −30 bp upstream of the junctions but accumulated on the exon sides (Figure 7B). This distribution profile of RNA polII mimics the nucleosome densities surrounding the exon-intron and intron-exon junctions in *Drosophila* [35], implicating an influence of chromatin structure on polII elongation. Our results support the hypothesis that nucleosomes enriched in exons function as ‘speed bumps’ at splicing junctions to slow the rate of RNA polII elongation in favor of RNA splicing [35]. To gain further insight on the uneven distribution of polII at splicing sites, we calculated the numbers of exons and splicing variants for genes manifesting the polII slowing in exons (254 genes in total) and compared to those of remaining genes. As expected, those genes with polII slowing in exons have 2.07 annotated splicing variants on average, which are significantly more than other *Drosophila* genes (Figure 7C and 7D).

## Discussion

In this paper, we have reported the development of a modified ChIP-Seq method and its application to construct a high-resolution whole-genome map of chromatin modification in *Drosophila*, which may represent an epigenetic landscape shared by all adult *Drosophila* cell types. Because our analysis involves diverse types of cells, it is not possible to distinguish when two chromatin marks are on the same DNA molecule and when they are in different cells. In fact, this issue exists for all types of ChIP analyses, including those using a homogenous cell line, which still have multiple stages of the cell cycle with different chromatin modifications. Despite this caveat of ChIP analysis, our analysis in whole flies is consistent with studies for *Drosophila* at major developmental stages and in cell lines [5], [6], [8]. Our work reveals fundamental features of chromatin modifications that are likely conserved among diverse organisms.

### Transposons contain both heterochromatic and euchromatic marks and are transcriptionally competent

Transposons occupy approximately one third of the *Drosophila* genome [36]. In the everlasting competition with these parasitic DNA, flies have evolved defensive mechanisms to regulate transposition of transposons. Recent discoveries indicate that transposon mobilization is controlled at two levels: transcriptional silencing by heterochromatin formation and post-transcriptional silencing via small RNA-based transposon RNA degradation. Our finding that heterochromatic marks H3K9me3 and HP1a are enriched in transposons indicates that a general scheme of transposon silencing in *Drosophila* is packaging the transposon-rich sequences into heterochromatin. Within heterochromatin, methyltransferase SU(VAR)3–9 sets the H3K9me3 mark, which recruits HP1a to initiate the heterochromatin formation [19], [20], [37]. In line with this view, we observed significant correlation between H3K9me3- and HP1a-levels in transposons. The most striking correlation is between H3K9me3 and H3K27me3, which suggests the possible co-localization of these two silencing marks in transposons. The co-localization of H3K9me3 and H3K27me3 has been observed in the chromocenter core regions on *Drosophila* polytene chromosomes [38]. Since no known enzyme can methylate H3 to trimethylation states for both Lysine 9 and Lysine 27, it would be interesting to investigate in the future whether SU(VAR)3–9 and E(Z) function synergistically to silence transposons by heterochromatin formation. Recently, RNA-based transposon silencing mechanisms have been uncovered. In *Drosophila*, posttranscriptional silencing pathways mediated by endo-siRNAs and piRNAs are involved in transposon silencing in the soma and germline, respectively [39]. A common scheme in these pathways is that transcription from transposon-rich regions is employed by host cells to generate defensive small RNAs, which in turn are utilized to degrade transposon transcripts. The presence of transposon-derived small RNAs dictates that transcriptional activity must exist in transposon sequences. In support of this idea, we find euchromatic mark H3K4me3 is indeed prevalent in some but not all transposons. This observation also echoes our previous finding that a transposon-rich region in the subtelomere of the right arm of chromosome 3 (3R-TAS) contains both heterochromatic (H3K9me2, H3K9me3 and HP1a) and euchromatic (H3K4me2, H3K4me3 and H3K9ac) marks [16]. Interestingly, this well-defined heterochromatin region is transcriptionally competent, giving rise to a panel of piRNAs and permissive to transcriptional activities from a reporter gene inserted in this region. Therefore, it is conceivable that many transposons and repetitive sequences with similar epigenetic states are also transcriptionally active, albeit at low levels.

### Histone code and RNA polII level/position around the TSS predict the gene expression level as well as the functionality of these genes

Our analysis of protein-coding genes reveals three salient features of chromatin modifications, which reflect distinct histone codes, in all transcriptionally active genes.

First, their transcribed regions are all enriched with H3K9ac. This is consistent with the notion that H3K9ac specifically associates with transcriptional activity and can spread over the whole gene body.

Second, the TSSs and TxEnds of active genes are further enriched with H3K4me3 (Figure 3F), which is consistent with the enrichment of H3K4me3 around TSSs of transcriptionally active genes in mammalian genomes. In addition, the drastic enrichment of H3K4me3 and H3K9ac in the 5′ transcribed region (5′TR) and the sharp decrease at TSSs to become severely under-represented in promoter regions (Figure 3F and 3G) is also observed for H3K4me3 in the human genome. In contrast to H3K4me3 and H3K9ac, H3K9me3 shows the opposite pattern in the promoter-5′TR; whereas H3K27me3 is underrepresented in both promoter and 5′TR of active genes (Figure 3H and 3I). These striking patterns of histone code around the TSS collectively represent an epigenetic signature for all actively transcribed genes. The robustness of this signature corresponds nicely to the transcriptional activity of a gene. It indicates that, in active genes, the promoter region is highly enriched in HP1a, the 5′TR is highly euchromatic, and the Polycomb repressive complex 1 is absent from both promoter and 5′TR of active genes.

Third, RNA polII is over-represented in both promoter and 5′TR of active genes. Moreover, the extent of the over-representation strictly corresponds to the transcriptional activity of the gene.

Fourth, stalled and elongating RNA polymerase II are positioned at +35 bp and +45 bp, respectively (Figure 5C), as discussed in detail in the next section.

Lastly, different active genes with different biochemical functions have distinct signatures of histone code at their TSS region. This finding, based on clustering 7,826 *Drosophila* genes according to similarities of their epigenetic profiles around TSSs, indicate the possibility that genes of similar functions are transcriptionally co-regulated by the same histone code set. This type of transcriptional regulation would be conceptually similar to the trans-operon fashion of translational regulation, where many mRNAs sharing common 3′UTR elements are regulated by a common translational repressor [40].

### RNA polII stalling as a mechanism of transcriptional regulation

Although it has been assumed that transcription initiation is the rate-limiting step in gene expression regulation, recent genome-wide mapping of RNA polII have revealed polII stalling as a critical control mechanism of gene expression during development [30], [31], [41]. In humans and fruitflies, RNA polII initiates on most genes but pauses immediately downstream of TSSs before it enters into productive elongation to generate full-length mRNAs. The permanganate footprinting has mapped the transcription bubbles between +30 bp to +80 bp on a small set of polII-stalled genes in *Drosophila* [30], [31]. Similarly, RNA-Seq of nuclear capped short RNAs has implicated that polII stalls within a region ranging from +25 bp to +60 bp downstream of TSSs [42]. In this study, we directly map RNA polII around TSSs by ChIP-Seq in an unprecedented 5-bp resolution, which unambiguously pinpoints the stalled polII in a 50 bp region, centered at +35 bp downstream of 685 TSSs. The fact that our ChIP-Seq sample is from whole flies indicates that polII stalling around +35 bp is a general mechanism ubiquitously present in most, if not all, cells.

In addition, we also found a lower but evident level of polII stalled around +45 bp on actively transcribing genes. Intriguingly, the different positions of stalled polII and elongating polII echo the different positions of +1 nucleosome at the 5′ ends of genes. In mammals, active genes (mostly with elongating polII) have the 5′ ends of the +1 nucleosome predominantly peaked at +40 bp, which is in contrast to the +10 bp positioning of +1 nucleosome in the inactive promoters (including promoters with stalled polII) [33]. In *Drosophila*, the predominant arrangement of the 5′ ends of the +1 nucleosome at +62 bp might allow the free access to the TSS by RNA polII at the initiation stage whereas also pose potential blockage downstream of TSS after initiation [43]. Thus, this 10 bp difference of RNA polII may reflect the influence of +1 nucleosome on stalled and elongating polII. It would be interesting to understand the positioning of +1 nucleosomes for polII-stalled genes and polII-elongating genes in the future. Alternatively, this difference of polII position suggests that RNA polII stalling may process in multiple steps.

### The dual functions of HP1a in transcriptional regulation

In addition to its well-known role in heterochromatin formation in transposon-rich regions, HP1a has been reported to positively regulate the expression of protein coding genes [44], [45], [46], [47]. This euchromatic function of HP1a is supported by both genome-wide mapping of HP1a binding sites [11], [12], [48] and gene expression analysis of HP1a mutants [29], [49]. By ChIP-Chip assays, HP1a is revealed to bind to the whole transcription unit, particularly exons, of its target genes [11], [48]. However, it remains controversial whether HP1a is associated with promoters in general [11], [48]. Our high-resolution mapping of HP1a by ChIP-Seq reveals the prevalence of HP1a binding to both promoters and transcription units of many protein-coding genes throughout the genome. In addition, the striking positive correlation between the accumulation of HP1a and the expression levels of its target genes strongly suggests a direct role of HP1a in transcriptional regulation. Such a correlation was previously observed for HP1a target genes on 4^th^ chromosomes and led to a “buffering” hypothesis, wherein HP1a and Painting of Fourth (POF) represent counteracting repressing and stimulating factors to achieve a stable expression of their common target genes [11]. Given the specific localization of POF, the function of HP1a in gene expression regulation on other autosomes remains elusive. Instead, our high-resolution map of chromatin modifications at 5-bp resolution, reveals an amazing similarity between HP1a localization and that of RNA polII on protein coding genes. Although we cannot exclude the possibility that HP1a and RNA polII locate separately in the same set of genes but in different cells, the almost identical spatial and quantitative distributions of HP1a and RNA polII strongly suggests that these two factors actually are co-localized. In support of this view, HP1a has been recently demonstrated to bind to mRNAs and interacts with RNA polII in *Drosophila* [46]. Our data further indicate that HP1a may co-localize with stalled polII on chromatin immediately downstream of TSSs, implicating a regulatory function of HP1a in controlling RNA polII elongation. This is consistent with our observation that HP1a is preferentially concentrated at TSSs of its regulated genes. We hypothesize that HP1a may function to stabilize RNA polII in its permissive state, waiting for external signals. In the absence of HP1a, un-stabilized polII will either terminate the transcription or prematurely transit to the elongating step. This hypothesis can potentially explain the profound opposing effects of HP1a on its activated and repressed genes, wherein a comparable amount of HP1a is observed around TSSs.

### Slowing down of transcriptional elongation at splicing junctions

Perhaps, the most unexpected finding in our study is that RNA polymerase II is concentrated on the exon sides of exon-intron and intron-exon junctions. This enrichment clearly indicates that elongating polII moves at a reduced rate within exons. These data provide strong direct evidence to a proposed kinetic model, which suggests that a reduced transcriptional elongation rate may facilitate the recognition of splice sites by the transcription-coupled splicing machinery [50]. This model is further supported by recent studies, which clearly demonstrated nucleosomes and trimethyl Histone 3 at Lysine 36 (H3K36me3) are preferentially enriched in exons comparing to introns [35], [51], [52], [53], [54]. This kinetic model further predicts that genes with multiple exons and/or alternative splicing events should preferentially demand polII slowing at their splicing junctions. Indeed, we found genes manifesting RNA polII slowing at exon-intron junctions have significant more RNA splicing variants than other genes. Taken together, our findings, along with a series of recent discoveries, support that the positioning of modified histones and nucleosomes marks the exons to slow down RNA polII elongation. This slowing down of RNA polII may facilitate the recruitment of splicing machinery to recognize *cis*-acting regulatory elements on emerging nascent RNA [35]. Notably, we cannot exclude the possibility that the depletion of RNA polII centered at −30 bp of intron-exon junctions is due to the polypyrimidine tract localized at this region. Such polypyrimidine tracts, predominant with thymidine (T) repeat and thus lack of uniqueness, may cause bias during micrococcal nuclease digestion and during mapping of sequencing tags to the genome. Further investigations based on different techniques may shed light on the precise polII processivity at intron-exon junctions.

## Materials and Methods

### Isolation of nuclei

Approximately 5 ml of newly eclosed flies (*w^1118^*) were collected, frozen in liquid nitrogen, and pulverized to a fine powder using a mortar and pestle. The fine powder was resuspended in 5 ml of Buffer A+ [60 mM KCl, 15 mM NaCl, 13 mM EDTA pH 8.0, 0.1 mM EGTA, 15 mM HEPES pH 7.4, 0.5 mM DTT, 0.5% NP-40, 1× Protease Inhibitor Cocktail (1xPI, Roche)] and sequentially disrupted with ∼10 strokes in a 7 ml homogenizer (Dounce) and ∼20 strokes in a 15 ml homogenizer (Wheaton). The homogenate was then filtered through two layers of Miracloth, loaded onto 2 ml of Buffer AS (60 mM KCl, 15 mM NaCl, 1 mM EDTA pH 8.0, 0.1 mM EGTA, 15 mM HEPES pH 7.4, 0.3 M sucrose) and centrifuged at 3,500 rpm for 12 minutes at 4°C (Beckman Coulter Optima L-100 XP Ultracentrifuge). The cytosolic layer was removed and the nuclei pellet were resuspended in 5 ml of Buffer A (60 mM KCl, 15 mM NaCl, 1 mM EDTA pH 8.0, 0.1 mM EGTA, 15 mM HEPES pH 7.4, 0.5 mM DTT, 1 mM PMSF, 5 mM Sodium Butyrate, 1xPI). The nuclei solution was transferred to a 7 ml homogenizer (Wheaton) and disrupted with ∼10 strokes of the “loose” pestle. The solution was again loaded onto 2 ml Buffer AS and centrifuged at 3,500 rpm for 10 minutes at 4°C. The crude nuclei were aliquoted into 2 ml siliconized eppendorf tubes and stored at −80°C until ∼5×10^9^ nuclei were collected.

### Micrococcal nuclease digestion

The nuclei samples were thawed on ice and pooled. The volume was adjusted with Buffer AC (60 mM KCl, 15 mM NaCl, 0.1 mM EGTA, 15 mM HEPES pH 7.4, 0.5 mM DTT, 1 mM PMSF, 5 mM CaCl2, 5 mM Sodium Butyrate, 1xPI) so that the final ratio of nuclei to Buffer AC equals 8∶3. Pilot experiments determined that 0.1 U micrococcal nuclease (MNase, USB Corp.) will digest 120 µg chromatin DNA and produce mono-, di-, and poly-nucleosomes in a 5 minute reaction. DNA concentrations of the nuclei solution was determined by A_260_ absorbance readings after alkali lysis. 0.1 U MNase was added into nuclei based on DNA concentration and incubated at 37°C for 5 minutes. 10 µl 0.5 M EDTA (pH 8.0) was added to stop the digestion. The nuclei pellets were resuspended in 500 µl Buffer AG (60 mM KCl, 15 mM NaCl, 10 mM EDTA pH 8.0, 0.1 mM EGTA, 15 mM HEPES pH 7.4, 0.5 mM DTT, 5% Glycerol, 5 mM Sodium Butyrate, 1xPI), centrifuged at 5,000 rpm. Supernatant containing mono- and di-nucleosomal fraction was separated from the pellets (poly-nucleosomal fraction). This extraction was repeated 3 times in total. The pellets after the last extraction were resuspended in 500 µl Buffer AG and pooled together. The polynucleosomal fraction was sonicated ∼30 times of 20 seconds pulses at 30% output (Branson Sonifer 450 with a microtip).

### Native chromatin immunoprecipitation (N–ChIP)

The mono-, di-nucleosomal fraction and the sonicated poly-nucleosomal fraction were pre-cleared with Protein A Sepharose beads (Millipore) for 1 hour with constant agitation. The fractions were then aliquoted into 15 ml siliconized tubes. The histone antibodies were then added to each tube: 50 µg anti-trimethyl-H3K9 (Upstate), 25 µg anti-trimethyl-H3K27 (Upstate), 50 µg anti-acetyl-H3K9 (Upstate), 50 µg anti-trimethyl-H3K4 (Upstate), 25 µg anti-Histone H3 (Upstate). Chromatin was incubated with antibodies overnight at 4°C with rotation. The ChIP beads were equilibrated overnight with tRNA in Buffer AG (200 µg tRNA/250 µl beads) and washed with Buffer AG for 3 times to remove any excess tRNA. The tRNA-coated beads (5 µl dry beads per1 µg antibody) were added to each sample and incubated for 2 hours at 4°C with rotation. The beads were washed sequentially with Wash Buffer 1 (60 mM KCl, 15 mM NaCl, 10 mM EDTA pH 8.0, 0.1 mM EGTA, 15 mM HEPES pH 7.4, 0.5 mM DTT), Wash Buffer 2 (60 mM KCl, 55 mM NaCl, 10 mM EDTA pH 8.0, 0.1 mM EGTA, 15 mM HEPES pH 7.4, 0.5 mM DTT), and Wash Buffer 3 (60 mM KCl, 105 mM NaCl, 10 mM EDTA pH 8.0, 0.1 mM EGTA, 15 mM HEPES pH 7.4, 0.5 mM DTT). After washing, beads were incubated in 1 ml Elution Buffer (50 mM TrisHCl pH 9.0, 20 mM EDTA, 1% SDS) at room temperature for 1 hour. The supernatants were phenol/chloroform extracted in PhaseLocking gel and ethanol precipitated. The precipitated DNA pellets were submitted for Illumina library construction and sequencing.

### Crosslinking chromatin immunoprecipitation (X–ChIP)

Formaldehyde was added to a final concentration of 0.1% to the nuclei and incubated at room temperature for 15 minutes. To quench the crosslinking, 2.5 M glycine was added to the nuclei to a final concentration of 0.125 M. Quenching was performed at room temperature for 10 minutes with constant agitation. Pellet nuclei were combined and resuspended into 20 ml ChIP Buffer (75 mM NaCl, 50 mM HEPES pH 7.4, 1 mM EDTA, 1 mM DTT, 5 mM MgCl_2_, 1XPI, 10% Glycerol) in a 50 ml siliconized tube. The nuclei were sonicated on ice for ∼30 second intervals at 30% output for 2 hours. 250 µl Protein A Sepharose beads were used for every 5×10^8^ nuclei. The beads were washed with ChIP Buffer and then equilibrated with tRNA (200 µg/250 µl beads) in ChIP Buffer + 1% BSA for overnight at 4°C. The beads were then washed with ChIP buffer for 3 times to remove any excess tRNA. Antibodies were added and incubated with beads overnight at 4°C: 250 µl crude anti-HP1a antisera (Covance), 160 µg anti-RNA Polymerase II (clone CTD4H8, Millipore), 200 µg mouse IgG1 (for mock ChIP, Abcam). The beads were then washed with ChIP buffer to remove any excess antibody. Chromatin samples were incubated with antibody-bound beads overnight at 4°C. The beads were washed for 5 minutes for each washing buffer: 1. RIPA 150 (50 mM HEPES pH 7.4, 150 mM NaCl, 2 mM EDTA pH 8.0, 1% Triton X-100, 0.1% SDS), 2. RIPA 500 (50 mM HEPES pH 7.4, 500 mM NaCl, 2 mM EDTA, 1% Triton X-100, 0.1% SDS), 3. LiCl Buffer (50 mM HEPES pH 7.4, 0.25 M LiCl, 1 mM EDTA, 1% NP-40), 4. TE Buffer. The beads were then eluted at room temperature in 500 µl Elution Buffer for 30 minutes. The supernatant was transferred to another tube and the elution was repeated with another 500 µl Elution Buffer. 8 µl Proteinase K (20 mg/ml) was added to the 1 ml combined elute samples and incubated at 37°C for 30 minutes. The ChIP samples were then reversed crosslinked by incubating at 65°C for 4 hours, phenol/chloroform extracted in PhaseLocking gels, and ethanol precipitated. The ChIP DNA pellets were dissolved in TE. Adapters are ligated to the ends of the ChIP DNA and PCR amplified before sequencing on Illumina GA.

### Bioinformatic analysis of ChIP–Seq

Sequenced 35 nt reads (with <5 ambiguous bases) and corresponding quality tracks were collected from the Bustard module of Illumina Analysis Pipeline and transformed into FASTQ format. A PERL-coded Illumina ChIP-Seq analysis pipeline was developed to streamline the tag mapping and downstream data collection and statistic analyses. Input reads were iteratively mapped to the *Drosophila melanogaster* genome (BDGP R5) by a third-party SOAP program with increasing allowance of mismatches (up to 5 bases) and indels (up to 4 bases), until the majority (>60%) of input reads were mapped to the reference genome. Both unique-mapping tags (mapped to only one genomic locus) and multiple-mapping tags (mapped to more than one genomic loci) were retained and only the best genomic matching site(s) were reported. Numbers of tags (mapped Illumina reads) for each ChIP-Seq library are listed in Table S1.

Because Illumina only sequences the first 35 nucleotides from the 5′ ends of DNA fragments, we applied a previously published tag extension approach (XSET) to score the genome. Briefly, a scoring matrix, reflecting the probabilities of the length of precipitated DNA fragments, was determined by the intensities of the input DNA on an agarose gel (Figure S1B). These probability scores were employed to indicate the relative possibilities of associations of epigenetic marks/regulators with target genomic regions. Scores of ChIP-Seq tags were allocated into 50 bp bins across the entire genome (including euchromatic arms, sequenced internal scaffolds and unmapped regions). For each tag, the genomic location of the 5′ end determines the first bin. The probability scores were given to the first bin and the downstream 9 bins (Figure S1C). In order to generate comparable scores for different ChIP datasets, raw scores were transformed via three normalizations (Figure S1D–S1G). First, accumulated raw scores from all tags were normalized as to 10 million tags were sequenced (Figure S1D). To subtract scores of control datasets from epigenetic mark datasets (hereafter called experimental datasets), we further normalized scores based on non-specific noise levels in all ChIP-Seq datasets (Figure S1E). To this end, normalized scores (per 10 million tags) were plotted against corresponding bin numbers for each experimental dataset and the control dataset (as exemplified by RNA polII ChIP-Seq vs. mock ChIP-Seq in Figure S1F). A critical value, beyond which the corresponding bin numbers in an experimental dataset are always more than those in the control dataset, was determined for each experimental/control dataset pair (Figure S1F). A normalizer was further determined for each experimental/control dataset pair in a way that the correlation coefficient between these two datasets for values lower than the critical value are maximized when the scores of the experimental dataset are multiplied by this normalizer. We found this normalizer can be estimated by the ratio between X (representing the peak value of the control dataset) and Y (representing the peak value of the experimental datasets) for most datasets examined (Figure S1F). We calculated adjusted scores for all bins in experimental datasets with signals from control datasets subtracted (Figure S1G). For an adjusted score, we estimated the FDR as the ratio of the number of bins that the control dataset indicated should occur by chance, to the number observed in experimental dataset. For each experimental dataset, we chose a threshold of adjusted scores as the smallest adjusted scores that was equivalent to FDR<0.001. Only adjusted scores above this threshold were reported to indicate the relative abundance of epigenetic marks across the genome. For each ChIP-Seq dataset, the final adjusted ChIP-Seq scores were recorded into files in wiggle track format (WIG) and browser extensible format (BED) for viewing the data in Integrated Genome Browser (Affymetrix) and the UCSC Genome Browser.

### ChIP–Seq correlation analyses with ChIP–Chip and DamID–Chip

HP1a ChIP-Chip datasets were collected from Gene Expression Omnibus (GEO) database (GSM205826, GSM205827 and GSM205828). HP1a DamID-Chip datasets were collected from GEO database (GSM151831, GSM151832 and GSM151833). Genome coordinates from both datasets were changed to the *Drosophila melanogaster* genome (BDGP R5) by UCSC liftover tools. To correlate ChIP-Chip with ChIP-Seq, the genomic regions interrogated by Nimblgene tiling array (GPL5404) were divided into 1-kb windows sorted based on their ChIP-Chip scores for HP1a. Windows were grouped into 100 percentiles and the corresponding averaged ChIP-Seq (U) scores were calculated. Pearson Product-Moment correlation was performed between ChIP-Chip scores and ChIP-Seq (U) scores for these 100 percentiles. To correlate DamID-Chip with ChIP-Seq, the genomic regions interrogated by Nimblgene tiling array (GPL2678) were divided into 1-kb windows sorted based on their DamID-Chip scores for HP1a. Windows were grouped into 100 percentiles and the corresponding averaged ChIP-Seq (U+M) scores were calculated. Pearson Product-Moment correlation was performed between ChIP-Chip scores and ChIP-Seq (U+M) scores for these 100 percentiles.

### Gene expression analysis

Gene expression information of adult whole flies was obtained from Gene Expression Omnibus (GSE5382: GSM106918, GSM122994, GSM123002, GSM123003, GSM123007 and GSE7763: GSM188112, GSM188113, GSM188114, GSM188115). In total, 12,523 genes were interrogated by Affymetrix *Drosophila* GeneChip 2.0 microarray assays in both datasets. Of these, 6,756 genes show consistent relative expression levels (denied by SAM analyses) between samples and between datasets. These 6,756 genes were sorted based on the averaged expression values from microarray replicates, and were further classified either into 10 gene expression groups or 100 gene expression percentiles by ranks in the expression profile.

Gene expression information of wild type and HP1a RNAi third instar larva was downloaded from GEO (GSM67069, GSM67070, GSM67067, GSM67068, GSM67073, GSM67071 and GSM67072). In total, 12,521 genes were interrogated by Affymetrix *Drosophila* Genome 2.0 Array. These genes were sorted and grouped into 100 percentiles based on the ratios of their expression in HP1a RNAi samples versus wild type samples. Gene expression of males and females were calculated separately. The correlation analyses shown in Figure 4C were performed using gene expression data from females. Although HP1a was shown to have male-specific effects on lethality and gene expression regulation, the analyses using gene expression data from males revealed essentially the same trends as those of females (data not shown).

### Alignment of ChIP–Seq scores around TSS, gene body, TxEnd, exon-intron junction, intron-exon junction, and correlation of ChIP–Seq scores to gene expression levels

Genomic coordinates of TSS, TxEnd, Exon-Intron junctions, Intron-Exon junctions were collected from *Drosophila melanogaster* annotation database R5.5 (ftp://ftp.flybase.net/releases). Genes in different expressive groups were aligned at the same direction at the transcription start sites (TSSs), at the mid points of gene bodies and the transcription end points (TxEnds), respectively. To avoid noises from nearby transposon/repetitive sequences, ChIP-Seq (U) scores were employed in this analysis. Sliding windows of 5 bp were applied in the calculation. For each 5-bp window, scores of all genes within a gene expression group were averaged after trimming off outliers (10% of the total gene number) in both ends. If a gene has more than one annotated TSSs or TxEnds, averaged scores of all TSS and TxEnd were included in the gene expression group that the gene belongs to.

### RNA polymerase II stalling analysis

To classify genes into groups with elongating RNA polII, stalled RNA polII and no polII, RNA polII stalling index was calculated in the same way as previously published [31], [33]. To avoid the noises from nearby transposon/repetitive sequences, ChIP-Seq (U) scores were used in this analysis. The polII scores of promoters were calculated as the average RNA polII ChIP-Seq scores within TSS-surrounding regions (−500 bp∼+500 bp). The polII scores of gene bodies were calculated as the average RNA polII scores within gene bodies downstream of TSSs (+750 bp∼+2500 bp). If the promoter region or gene body region of two genes were overlapping with each other, scores from overlapped regions were not included in the calculation. A stalling index was then defined as the ratio of the promoter polII score over the gene body score. Genes with elongating polII were defined as those with promoter scores at least 5 and with stalling index less than 3. Genes with stalled polII were defined as those with promoter scores at least 5 and with stalling index greater than 10. Genes without polII were defined as genes with both promoter score and body score less than 1. To infer the precise positions of RNA polII at TSSs, Illumina reads from polII ChIP-Seq were mapped to the TSS surrounding regions (−1 kb∼+1 kb) and separated based on their relative orientation to genes. The number of Illumina reads in each 5-bp windows surrounding TSSs were normalized to the corresponding read numbers from the mock ChIP-Seq. A positive value for normalized read number indicates RNA polII is enriched whereas a negative value indicates RNA polII is depleted. Similar analysis was performed to investigate RNA polII pausing at exon-intron and intron-exon junctions.

### Estimation of quantitative gene expression levels by artificial neural network

Dynamic neural network with an input layer (10 neurons in analysis shown in Figure 6B, or 114 neurons in analysis shown in Figure 6C), two hidden layers (2 neurons by 3 neurons) and an output layer (quantitative gene expression estimation) were constructed and trained by 50% of random selected input data. To infer the relative important of each input variables, 10 runs of independent training/estimation were performed (Figure 6B). Relative importance was calculated from neuron weights and averaged.

### Gene ontology analyses

Flybase IDs of a gene set were analyzed by a web-based Functional Annotation Tool of Database for Annotation, Visualization and Integrated Discovery (DAVID, http://david.abcc.ncifcrf.gov/).

## Supporting Information

Figure S1 A new ChIP-Seq analysis method for whole-genome mapping of chromatin modifications. (A) Ethidium Bromide staining of input genomic DNA, which was purified from euchromatic fractions and heterochromatic fractions (after sonication) of wild type whole flies (left panel). Gel image was taken and analyzed by Kodak Gel Logic 200 imager. Intensity profiles of input DNA (combined both euchromatic and heterochromatic fractions) and molecular weight markers are shown (right panel). (B) Specificity test of HP1a antibody from Covance. Only one predominant band was recognized for whole cell lysates made from HP1a wild type (*w^1118^*) adult flies and third instar larva. The band was absent from whole cell lysate made from a HP1a mutant [Su(var)205]. GAPDH was blotted as a loading control. (C) The scoring matrix deduced from the intensity profile of input DNA and used for scoring genome-wide bins. (D) A schematic view of how probability scores are employed to score the genome. A certain part of genome (horizontal bar) is divided into 50-bp bins (between two vertical bars). Two Solexa tags mapped to the forward strand (green) and a Solexa tag mapped to the reverse strand are giving scores to the 50-bp bins based on the score matrix shown in panel B. Accumulative probability scores are shown at below. (E) Accumulative raw scores are normalized based on the numbers of sequenced tags. (F) A schematic view of how to normalize ChIP-Seq datasets based on noise levels before background subtraction. (G) Raw scores of bins (X-axial) are plotted against numbers of bins (Y-axial) for RNA polII and mock ChIP-Seq datasets. The critical value is defined as the right-most crossing point of RNA polII curve and mock ChIP-Seq curve on this plot. The scores, which are smaller than this critical point, are considered as noises (shaded area). The peak value of mock ChIP-Seq curve (X) divided by the peak value of RNA polII curve (Y) can be used to estimate the background noise normalizer, which maximizes the correlation between two datasets for the noises (within shaded area). (H) The equation used to calculate adjusted scores (enrichment indexes).
(PDF)

Figure S2 A correlation analysis compares HP1a localization interrogated by ChIP-Seq and DamID-Chip analysis. 72,842 1-kb genomic bins were ranked into 100 percentiles by their scores in DamID-Chip analysis [12]. Average scores for bins within a percentile were shown in dots for both ChIP-Seq (yellow) and ChIP-Chip (red). Because the customized NimbleGene array used in the DamID-Chip assay includes probes for repetitive sequences, ChIP-Seq (U+M) scores were used in this comparison. Pearson Product-Moment correlation coefficient was calculated.
(PDF)

Figure S3 Distribution of H3K27me3 over the euchromatic genome of *Drosophila*. (A) Distribution of normalized H3K27me3 ChIP-Seq (U) scores over the euchromatic genome. Red bars denote predicted Polycomb/Trithorax response elements (PRE/TREs) [25]. Asterisks above red bars indicate PRE/TREs showing significant enrichment of H3K27me3 comparing to 20 groups of randomly selected intergenic regions with matched length (167 random regions per group). Blue bars represent matches to cytological binding sites of polycomb proteins on polytene chromosomes previously revealed by immunostaining [25] and/or in [23]. Green cytoband IDs above blue bars denote corresponding PcG/Trx binding sites are overlapping with both predicted PRE/TREs and enriched H3K27me3 marks. Yellow cytoband IDs below blue bars indicate PcG/Trx binding sites enriched for H3K27me3. Black ovals indicate the locations of centromeres. (B) Detailed views of H3K27me3-enriched regions over *Antennapedia* complex (*ANT-C*), *Bithorax* complex (*BX-C*) and a 200-kb region between *mod(mdg4)* and *InR*. Red arrow heads denote locations of predicted PRE/TREs [25]. (C) Predicted PRE/TREs are significantly enriched for H3K27me3 marks. H3K27me3 ChIP-Seq scores over 1.5 kb surrounding regions of the centers of 167 predicted PRE/TREs were averaged and plot for each 50 bp windows. Averaged H3K27me3 ChIP-Seq scores and 99% confidence intervals of scores for random regions were shown in deep blue dots and error bars. The numbers of predicted PRE/TREs in each 50-bp window are shown in green.
(PDF)

Table S1 Summary of Solexa Sequencing.
(PDF)

Table S2 H3K27me3-Enriched Genomic Regions.
(PDF)

Table S3 GO Term Enrichment Analysis of Gene Clusters.
(PDF)
