# Supplementary figures and images for: A High-Resolution Whole-Genome Map of Key Chromatin Modifications in the Adult Drosophila melanogaster
Source: PLoS Genet. 2011 Dec 15;7(12):e1002380. doi: 10.1371/journal.pgen.1002380 (PMC3240582; doi:10.1371/journal.pgen.1002380)

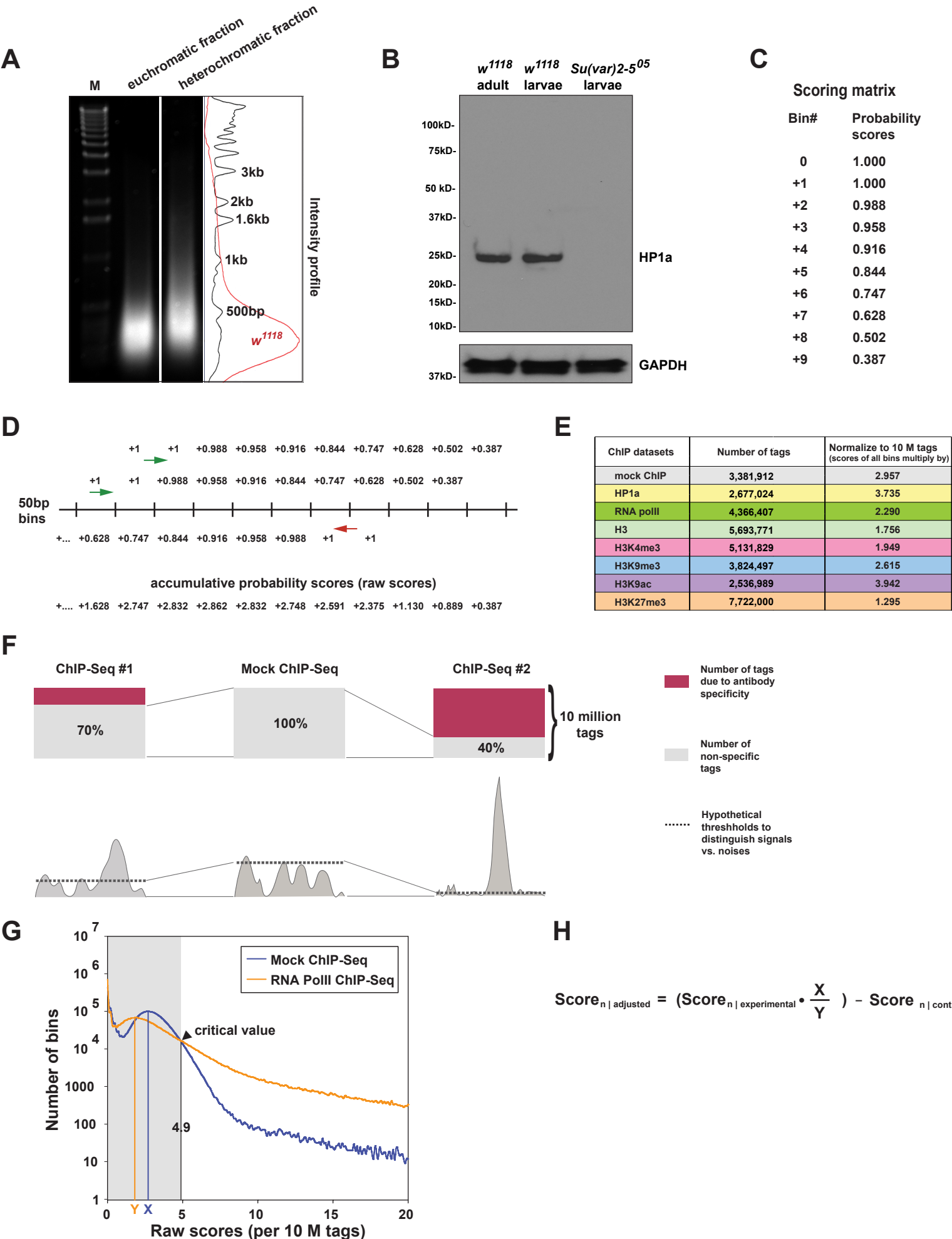

Supplementary Figure S1

Supplement: Figure S1 — A new ChIP-Seq analysis method for whole-genome mapping of chromatin modifications. (A) Ethidium Bromide staining of input genomic DNA, which was purified from euchromatic fractions and heterochromatic fractions (after sonication) of wild type whole flies (left panel). Gel image was taken and analyzed by Kodak Gel Logic 200 imager. Intensity profiles of input DNA (combined both euchromatic and heterochromatic fractions) and molecular weight markers are shown (right panel). (B) Specificity test of HP1a antibody from Covance. Only one predominant band was recognized for whole cell lysates made from HP1a wild type (w1118) adult flies and third instar larva. The band was absent from whole cell lysate made from a HP1a mutant [Su(var)205]. GAPDH was blotted as a loading control. (C) The scoring matrix deduced from the intensity profile of input DNA and used for scoring genome-wide bins. (D) A schematic view of how probability scores are employed to score the genome. A certain part of genome (horizontal bar) is divided into 50-bp bins (between two vertical bars). Two Solexa tags mapped to the forward strand (green) and a Solexa tag mapped to the reverse strand are giving scores to the 50-bp bins based on the score matrix shown in panel B. Accumulative probability scores are shown at below. (E) Accumulative raw scores are normalized based on the numbers of sequenced tags. (F) A schematic view of how to normalize ChIP-Seq datasets based on noise levels before background subtraction. (G) Raw scores of bins (X-axial) are plotted against numbers of bins (Y-axial) for RNA polII and mock ChIP-Seq datasets. The critical value is defined as the right-most crossing point of RNA polII curve and mock ChIP-Seq curve on this plot. The scores, which are smaller than this critical point, are considered as noises (shaded area). The peak value of mock ChIP-Seq curve (X) divided by the peak value of RNA polII curve (Y) can be used to estimate the background noise normalizer, which maximizes [file pgen.1002380.s001.pdf]

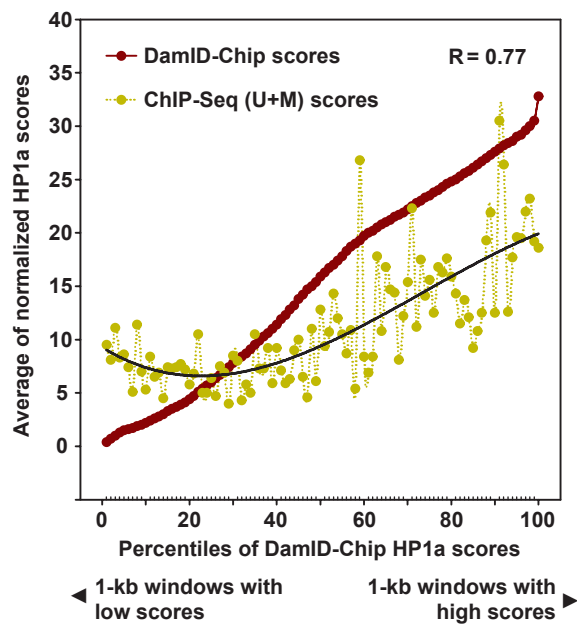

Supplementary Figure S2

Supplement: Figure S2 — A correlation analysis compares HP1a localization interrogated by ChIP-Seq and DamID-Chip analysis. 72,842 1-kb genomic bins were ranked into 100 percentiles by their scores in DamID-Chip analysis [12]. Average scores for bins within a percentile were shown in dots for both ChIP-Seq (yellow) and ChIP-Chip (red). Because the customized NimbleGene array used in the DamID-Chip assay includes probes for repetitive sequences, ChIP-Seq (U+M) scores were used in this comparison. Pearson Product-Moment correlation coefficient was calculated. (PDF) [file pgen.1002380.s002.pdf]

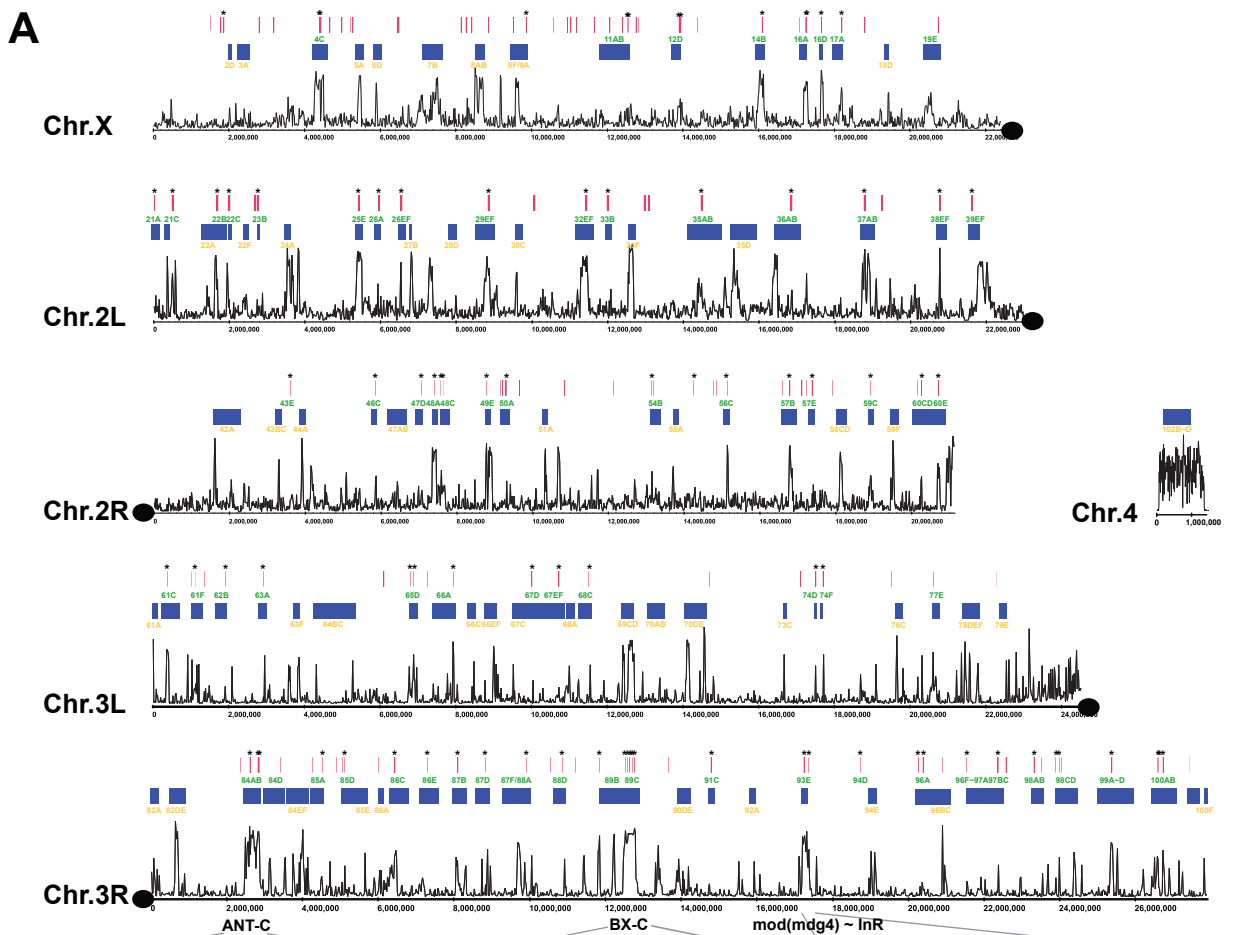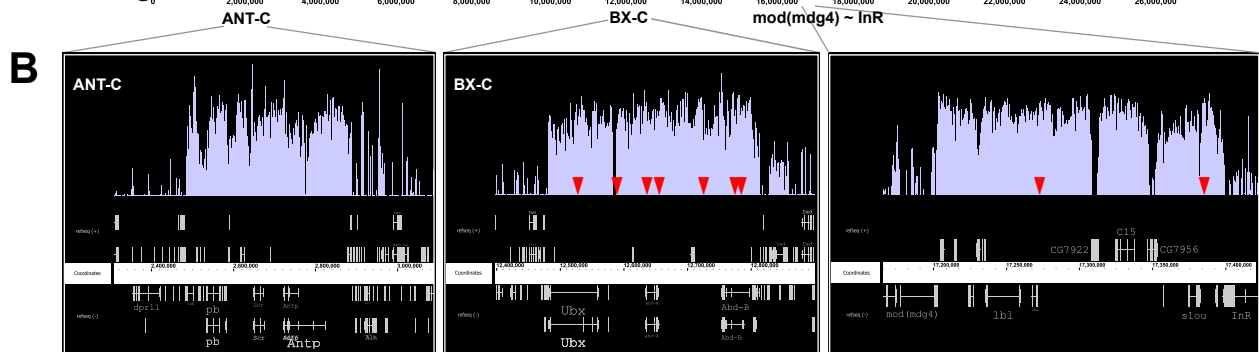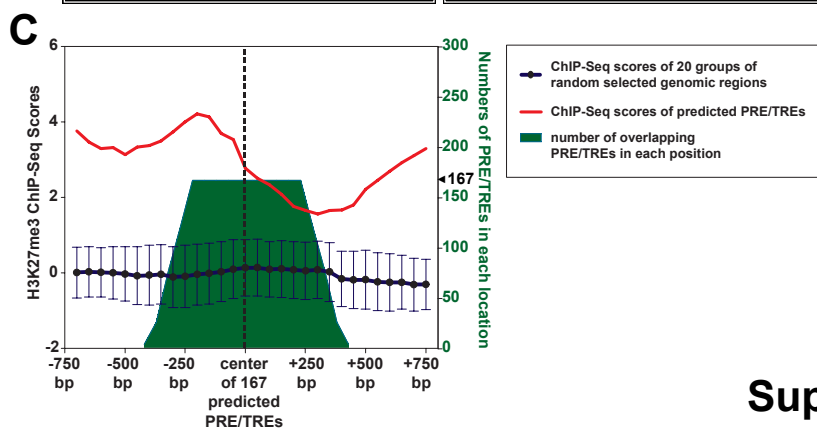

Supplementary Figure S3

Supplement: Figure S3 — Distribution of H3K27me3 over the euchromatic genome of Drosophila. (A) Distribution of normalized H3K27me3 ChIP-Seq (U) scores over the euchromatic genome. Red bars denote predicted Polycomb/Trithorax response elements (PRE/TREs) [25]. Asterisks above red bars indicate PRE/TREs showing significant enrichment of H3K27me3 comparing to 20 groups of randomly selected intergenic regions with matched length (167 random regions per group). Blue bars represent matches to cytological binding sites of polycomb proteins on polytene chromosomes previously revealed by immunostaining [25] and/or in [23]. Green cytoband IDs above blue bars denote corresponding PcG/Trx binding sites are overlapping with both predicted PRE/TREs and enriched H3K27me3 marks. Yellow cytoband IDs below blue bars indicate PcG/Trx binding sites enriched for H3K27me3. Black ovals indicate the locations of centromeres. (B) Detailed views of H3K27me3-enriched regions over Antennapedia complex (ANT-C), Bithorax complex (BX-C) and a 200-kb region between mod(mdg4) and InR. Red arrow heads denote locations of predicted PRE/TREs [25]. (C) Predicted PRE/TREs are significantly enriched for H3K27me3 marks. H3K27me3 ChIP-Seq scores over 1.5 kb surrounding regions of the centers of 167 predicted PRE/TREs were averaged and plot for each 50 bp windows. Averaged H3K27me3 ChIP-Seq scores and 99% confidence intervals of scores for random regions were shown in deep blue dots and error bars. The numbers of predicted PRE/TREs in each 50-bp window are shown in green. (PDF) [file pgen.1002380.s003.pdf]
