# Supplementary material for: A High-Resolution Whole-Genome Map of Key Chromatin Modifications in the Adult Drosophila melanogaster
Source: PLoS Genet. 2011 Dec 15;7(12):e1002380. doi: 10.1371/journal.pgen.1002380 (PMC3240582; doi:10.1371/journal.pgen.1002380)
Supplement: Table S1 — Summary of Solexa Sequencing. (PDF) [file pgen.1002380.s004.pdf]

Table S1. Summary of Solexa Sequencing

Sequencing Scale		Non-redundant Solexa tags			Solexa reads		
		total tag #	unique tag #	multiple tag #	total read #	unique read #	multiple read #
wild type flies	Mock ChIP	3,381,912	2,525,205	856,707	4,038,814	2,574,146	1,464,668
	H3	5,693,771	4,747,317	946,454	6,351,188	5,046,304	1,304,884
	HP1a	2,677,024	2,085,608	591,416	2,967,286	2,114,682	852,604
	RNA polII	4,366,407	3,861,312	505,095	5,626,556	4,805,130	821,426
	H3K4me3	5,131,829	4,234,712	897,117	5,650,921	4,339,650	1,311,271
	H3K9me3	3,824,497	2,970,904	853,593	4,543,831	3,157,785	1,386,046
	H3K9ac	2,536,989	2,355,671	181,318	3,054,914	2,556,881	498,033
	H3K27me3	7,722,000	6,176,152	1,545,848	8,979,461	6,392,159	2,587,302
	Total:	37,983,462	7.9 X coverage*		44,095,341		

Note*: 1 X coverage = 168,717,020 bp (total genome size) / 35bp (tag length) = 4,820,486 Solexa tags
