# Supplementary material for: A High-Resolution Whole-Genome Map of Key Chromatin Modifications in the Adult Drosophila melanogaster
Source: PLoS Genet. 2011 Dec 15;7(12):e1002380. doi: 10.1371/journal.pgen.1002380 (PMC3240582; doi:10.1371/journal.pgen.1002380)
Supplement: Table S2 — H3K27me3-Enriched Genomic Regions. (PDF) [file pgen.1002380.s005.pdf]

Table S2. H3K27me3-Enriched Genomic Regions

#	Chr	Start	End	Cytoband	Included genes
1	X	4,264,600	4,514,600	4C2 – 4C10	from *CG12688* to *pebbled*
2	X	5,404,145	5,509,399	5A2 – 5A7	from *CG12730* to *CG4136*
3	X	5,886,921	5,933,880	5D1 – 5D2	from *CG4766* to *roughex*
4	X	7,453,789	7,537,367	7B4 – 7B5	from *CG11369* to *cut*
5	X	8,499,811	8,571,154	7F8 – 8A1	from *caf1-180* to *CG12772*
6	X	8,615,374	8,729,815	8A3 – 8B2	*Lim1*
7	X	9,176,154	9,210,701	8D5 – 8D6	*lozenge*
8	X	9,578,305	9,672,630	8F9 – 9A1	from *buttonhead* to *CG1354*
9	X	15,955,928	16,142,413	14A8 – 14B5	from *Tob* to *disconnected*
10	X	17,199,002	17,312,249	16A1 – 16A5	from *CG5445* to *CG8611*
11	X	17,645,348	17,715,872	16C8 – 16D4	from *unc-4* to *Socs16D*
12	X	18,171,104	18,220,640	17A4 – 17A5	from *unpaired3* to *CG6023*
13	2L	365,460	403,965	21C1 – 21C2	from *aristaless* to *RNA polymerase I 135kD subunit*
14	2L	490,193	529,795	21D2 – 21E1	*u-shaped*
15	2L	576,945	602,773	21E2	from *PvuII-PstI homology 13* to *CG13689*
16	2L	1,618,334	1,704,688	22A3 – 22B1	*CG31666*
17	2L	1,942,442	1,974,262	22B6 – 22B8	from *CG33673* to *CG10908*
18	2L	3,530,332	3,706,345	24A4 – 24C1	from *drumstick* to *CG31778*
19	2L	3,813,067	3,862,929	24C5 – 24C8	*sloppy paired 1*, *sloppy paired 2*
20	2L	5,370,948	5,516,778	25D7 – 25E5	from *no mechanoreceptor potential C* to *CG14020*
21	2L	6,526,157	6,557,925	26E1 – 26E3	from *CG31637* to *osm-6*
22	2L	6,791,189	6,852,475	27B1 – 27C1	from *nrv nervana 2* to *Regulator of cyclin A1*
23	2L	7,276,810	7,383,183	27F1 – 27F3	from *wingless* to *neither inactivation nor afterpotential C*
24	2L	8,733,913	8,913,030	29E6 – 29F3	from *raw* to *CG34398*
25	2L	9,575,541	9,617,440	30B12 – 30C1	from *CG4389* to *GlcAT-S*
26	2L	11,285,629	11,502,330	32E2 – 32F2	from *Osiris 21* to *CG6488*
27	2L	12,546,538	12,688,682	33E9 – 33F3	from *bun* to *POU domain protein 2*
28	2L	14,371,166	14,598,857	35B1 – 35B3	from *pickpocket* to *CG3473*
29	2L	15,070,253	15,113,130	35C1 – 35C2	from *vasa* to *CG15269*

#	Chr	Start	End	Cytoband	Included genes
30	2L	15,271,526	15,502,007	35D1 – 35D2	from *CG15261* to *Translocase inner membrane 17*
31	2L	16,352,576	16,491,639	35F12 – 36A2	from *CaBP1* to *CG4580*
32	2L	18,722,205	18,938,718	37A1 – 37B6	from *CG15167* to *CG18397*
33	2L	20,766,705	20,788,538	38E8 – 38E10	from *diaphanous* to *Pomp*
34	2L	21,753,202	22,081,341	40A1– 40E2	from *CG31612* to *CG6691*
35	2R	1,592,648	1,641,028	41F8 – 41F9	from *vulcan* to *1(2)09851*
36	2R	3,294,313	3,323,158	43B2 – 43C1	from *CG11166* to *sine oculis*
37	2R	3,904,824	3,941,529	44A2 – 44A4	from *CG14762* to *CG12761*
38	2R	7,349,619	7,466,616	47F14 – 48A2	from *Enhancer of Polycomb* to *toutatis*
39	2R	8,759,535	8,804,006	49D6 – 49E1	from *CG3790* to *1(2)01424*
40	2R	8,831,603	8,932,240	49E3 – 49F1	from *Multi drug resistance 49* to *CG13323*
41	2R	10,321,885	10,396,090	51A2 – 51A4	from *phyllopod* to *CG17390*
42	2R	10,663,090	10,730,990	51C2 – 51C5	from *knot* to *Na^+-dependent inorganic phosphate cotransporter*
43	2R	15,130,374	15,165,327	56C4 – 56C6	from *coracle* to *ribbon*
44	2R	16,770,193	16,862,723	57B4 – 57B5	from *orthopedia* to *CG34115*
45	2R	18,104,965	18,183,183	58D2 – 58D3	from *CG30278* to *CG5819*
46	2R	19,454,116	19,554,762	59F1– 59F5	from *apontic* to *Phosphodiesterase 8*
47	2R	20,926,066	21,028,144	60F1 – 60F5	from *Neuropeptide-like precursor 1* to *Tyrosine kinase-related protein*
48	3L	365,771	434,930	61C1	from *trachealess* to *klarsicht*
49	3L	3,616,564	3,644,352	63E5	from *CG10357* to *CG12029*
50	3L	3,840,607	3,896,783	63F3 – 63F5	from *Arrowhead* to *Gustatory receptor 63a*
51	3L	6,754,926	6,987,284	65C4 – 65D6	from *CG32392* to *biniou*
52	3L	7,929,219	7,964,031	66A20 –66A21	from *Srp9* to *Ribonuclease X25*
53	3L	8,995,036	9,040,469	66F1 – 66F3	from *CG5087* to *CG5144*
54	3L	12,410,626	12,464,736	69B5 – 69C2	from *GTPase-activating protein 69C* to *CG10616*
55	3L	12,566,012	12,728,474	69C8 – 69D6	from *araucan* to *small nuclear ribonucleoprotein at 69D*
56	3L	14,076,125	14,183,875	70D2 – 70D3	from *CG8100* to *nanchung*
57	3L	14,555,711	14,612,135	70E2 – 70E4	from *CG34039* to *shade*
58	3L	19,636,298	19,678,333	76B11 – 76C1	from *Ornithine aminotransferase precursor* to *CG8765*
59	3L	20,533,576	20,712,097	77D1 – 77E3	from *CG4786* to *CG13253*

#	Chr	Start	End	Cytoband	Included genes
60	3L	21,442,007	21,477,597	78D2 – 78D3	from *Syntaxin 7* to *crocodile*
61	3L	21,568,985	21,602,830	78D7 – 78E1	from *CG32441* to *AP-2*
62	3L	21,758,442	21,818,290	78F1 – 78F3	from *Syntrophin-like 1* to *Cyclin H*
63	3R	646,523	732,329	82D6 – 82E2	from *CG14657* to *Germ line transcription factor 1*
64	3R	2,485,947	2,888,201	84A1 – 84B2	from *labial* to *CG1988*
65	3R	3,125,740	3,175,059	84D3 – 84D5	from *rotund* to *nuclear export factor 4*
66	3R	3,750,304	3,794,138	84E5 – 84E6	*doublesex*
67	3R	3,953,417	4,165,317	84E13 – 84F13	from *CG7891* to *CG9603*
68	3R	6,241,765	6,501,354	86B4 – 86C4	from *CG6345* to *CG6465*
69	3R	8,049,047	8,173,498	87B3 – 87B7	from *CG17404* to *CG10038*
70	3R	8,882,095	8,905,013	87D11	*single-minded*
71	3R	9,666,675	9,784,442	88A1 – 88A3	from *CG31495* to *Inositol polyphosphate 1-phosphatase*
72	3R	11,809,275	11,869,670	89A11 – 89B1	from *serpent* to *pannier*
73	3R	12,186,970	12,258,657	89B14 – 89B16	from *CG14880* to *CG31279*
74	3R	12,480,296	12,814,366	89D6 – 89E6	from *Ultrabithorax* to *CG18622*
75	3R	17,203,138	17,399,241	93D9 – 93E4	from *mod(mdg4)* to *Insulin-like receptor*
76	3R	18,953,981	19,174,298	94E1 – 94E13	from *hedgehog* to *pointed*
77	3R	20,901,945	21,014,952	96B20 – 96C4	from *CG11920* to *distal antenna*
78	3R	24,369,978	24,428,240	98D4 – 98D6	from *CG12413* to *Heterogeneous nuclear ribonucleoprotein at 98DE*
79	3R	25,328,898	25,417,783	99B2 – 99B4	from *Trc8* to *alphabet*
80	3R	25,511,345	25,542,461	99B7 – 99B8	from *Odorant-binding protein 99c* to *Odorant-binding protein 99b*
81	3R	26,426,900	26,919,136	100A1 – 100B2	from *CG2246* to *Sox100B*
82	4	502,307	565,644	102C1 – 102C4	*Asator*, *Zfh2*
83	4	715,271	796,484	102D4 – 102E1	from *Myoglianin* to *bent*
84	4	820,302	876,918	102E1 – 102E5	from *CG11231* to *CG11148*
85	4	978,663	1,029,391	102F4 – 102F5	from *CG32016* to *twin of eyeless*
86	4	1,103,365	1,133,637	102F8	from *Activin-β* to *shaven*
