# Supplementary material for: A High-Resolution Whole-Genome Map of Key Chromatin Modifications in the Adult Drosophila melanogaster
Source: PLoS Genet. 2011 Dec 15;7(12):e1002380. doi: 10.1371/journal.pgen.1002380 (PMC3240582; doi:10.1371/journal.pgen.1002380)
Supplement: Table S3 — GO Term Enrichment Analysis of Gene Clusters. (PDF) [file pgen.1002380.s006.pdf]

Table S3. GO Term Enrichment Analysis of Gene Clusters

Cluster A.

Category	GO term	Count	%	P-value	Fold of enrichment
SP_PIR_KEYWORDS	alternative splicing	121	7.4	8.60E-39	3.8
SP_PIR_KEYWORDS	membrane	152	9.3	3.70E-26	2.4
SP_PIR_KEYWORDS	Developmental protein	100	6.1	2.00E-25	3.1
SP_PIR_KEYWORDS	transmembrane	131	8	4.40E-19	2.2
GOTERM_BP	imaginal disc development	113	6.9	4.50E-17	2.2
SP_PIR_KEYWORDS	oxidoreductase	85	5.2	6.70E-16	2.6
SP_PIR_KEYWORDS	dna-binding	79	4.8	1.00E-15	2.7
GOTERM_BP	metamorphosis	100	6.1	3.30E-15	2.2
GOTERM_BP	larval development (sensu Amphibia)	100	6.1	3.30E-15	2.2
SP_PIR_KEYWORDS	signal	75	4.6	4.00E-15	2.7
GOTERM_BP	cell morphogenesis	127	7.8	5.10E-15	2
GOTERM_BP	imaginal disc morphogenesis	96	5.9	6.60E-15	2.2
GOTERM_BP	wing disc development	66	4	8.40E-15	2.7
SP_PIR_KEYWORDS	hydrolase	149	9.1	1.00E-14	1.9
SP_PIR_KEYWORDS	glycoprotein	71	4.3	1.20E-14	2.7
GOTERM_BP	organ morphogenesis	109	6.7	1.10E-13	2
GOTERM_BP	imaginal disc-derived appendage morphogenesis	64	3.9	1.90E-13	2.6
SP_PIR_KEYWORDS	Transcription regulation	62	3.8	4.30E-13	2.8
GOTERM_BP	wing disc morphogenesis	59	3.6	5.40E-13	2.7
GOTERM_BP	imaginal disc-derived wing morphogenesis	58	3.6	9.10E-13	2.7
SP_PIR_KEYWORDS	transferase	103	6.3	1.00E-12	2.1
GOTERM_BP	tissue development	81	5	2.00E-12	2.2
UP_SEQ_FEATURE	splice variant	106	6.5	3.30E-12	1.8
SP_PIR_KEYWORDS	Transcription	59	3.6	1.60E-11	2.6
SP_PIR_KEYWORDS	metal-binding	102	6.2	4.40E-11	2
SP_PIR_KEYWORDS	DNA binding	31	1.9	1.10E-10	3.9
SP_PIR_KEYWORDS	nucleus	100	6.1	4.70E-10	1.9
SP_PIR_KEYWORDS	nucleotide-binding	94	5.8	2.00E-09	1.9

SP_PIR_KEYWORDS	zinc	79	4.8	2.10E-09	2
GOTERM_CC	cell junction	30	1.8	4.70E-09	3.3
GOTERM_BP	gland development	48	2.9	6.30E-09	2.4
GOTERM_BP	salivary gland development	43	2.6	8.50E-09	2.5
SP_PIR_KEYWORDS	kinase	48	2.9	1.10E-08	2.5
GOTERM_BP	eye development	62	3.8	1.40E-08	2.1
GOTERM_BP	eye-antennal disc development	57	3.5	1.90E-08	2.2
GOTERM_CC	adherens junction	25	1.5	2.10E-08	3.5
GOTERM_BP	regulation of tube architecture, open tracheal system	20	1.2	2.20E-08	4.2
GOTERM_BP	ectoderm development	45	2.8	2.80E-08	2.4
GOTERM_BP	sensory organ development	72	4.4	3.00E-08	1.9
SP_PIR_KEYWORDS	cytoplasm	50	3.1	3.10E-08	2.3
GOTERM_BP	eye-antennal disc morphogenesis	55	3.4	3.50E-08	2.2
GOTERM_BP	establishment and/or maintenance of cell polarity	34	2.1	5.70E-08	2.7
GOTERM_BP	cell part morphogenesis	70	4.3	9.10E-08	1.9
GOTERM_BP	cell projection morphogenesis	70	4.3	9.10E-08	1.9
GOTERM_BP	cell projection organization and biogenesis	70	4.3	9.10E-08	1.9

Cluster B.

Category	GO term	Count	%	P-value	Fold of enrichment
GOTERM_MF	serine-type endopeptidase activity	9	5.2	4.10E-03	3.4
GOTERM_BP	proteolysis	12	6.9	5.70E-03	2.5
GOTERM_MF	steroid hormone receptor activity	3	1.7	7.90E-03	21.6

Cluster C.

Category	GO term	Count	%	P-value	Fold of enrichment
GOTERM_CC	cytoplasmic part	95	20.6	6.00E-10	1.7
GOTERM_CC	cytoplasm	106	23	9.20E-09	1.6
GOTERM_CC	cytosolic ribosome (sensu Eukaryota)	17	3.7	1.10E-08	6.1
GOTERM_CC	ribosomal subunit	22	4.8	2.00E-08	4.4
GOTERM_CC	macromolecular complex	88	19.1	4.80E-08	1.7
GOTERM_CC	ribosome	24	5.2	5.00E-08	3.8
GOTERM_MF	structural constituent of ribosome	24	5.2	5.10E-08	3.9

Cluster D.

Category	GO term	Count	%	P-value	Fold of enrichment
GOTERM_CC	cytosolic ribosome (sensu Eukaryota)	27	3	2.40E-12	5.2
GOTERM_CC	cytosolic part	28	3.1	4.40E-10	4.1
GOTERM_MF	protein binding	376	42.3	9.00E-09	1.2
GOTERM_CC	ribosomal subunit	30	3.4	3.10E-08	3.2
KEGG_PATHWAY	Ribosome	24	2.7	6.30E-08	3.4

Cluster E.

Category	GO term	Count	%	P-value	Fold of enrichment
GOTERM_BP	sensory perception of chemical stimulus	40	7.7	1.40E-23	7.4
GOTERM_MF	G-protein coupled receptor activity	45	8.7	2.20E-20	5.2
GOTERM_BP	G-protein coupled receptor protein signaling pathway	49	9.4	3.10E-18	4.3
GOTERM_CC	integral to membrane	71	13.7	1.30E-09	1.9
GOTERM_CC	intrinsic to membrane	71	13.7	1.40E-09	1.9

Cluster F.

Category	GO term	Count	%	P-value	Fold of enrichment
GOTERM_MF	oxidoreductase activity	37	10.3	6.80E-05	2
GOTERM_MF	oxidoreductase activity, acting on NADH or NADPH	9	2.5	9.30E-05	6.2
GOTERM_CC	intracellular organelle part	63	17.6	9.70E-05	1.5
